# Comparative transcriptome study of switchgrass (*Panicum virgatum* L.) homologous autopolyploid and its parental amphidiploid responding to consistent drought stress

**DOI:** 10.1186/s13068-020-01810-z

**Published:** 2020-10-15

**Authors:** Peilin Chen, Jing Chen, Min Sun, Haidong Yan, Guangyan Feng, Bingchao Wu, Xinquan Zhang, Xiaoshan Wang, Linkai Huang

**Affiliations:** 1 Department of Grassland Science, Animal Science and Technology College, Sichuan Agricultural University, Chengdu, 611130 China; 2 Institute for Biology, Plant Cell and Molecular Biology, Humboldt-Universität zu Berlin, 10115 Berlin, Germany; 3 Department of Horticulture, Virginia Tech, Blacksburg, VA 24061 USA

**Keywords:** *Panicum virgatum* L., Whole genome duplication, Drought response, MicroRNA, Transcriptome, WGCNA

## Abstract

**Background:**

Newly formed polyploids may experience short-term adaptative changes in their genome that may enhance the resistance of plants to stress. Considering the increasingly serious effects of drought on biofuel plants, whole genome duplication (WGD) may be an efficient way to proceed with drought resistant breeding. However, the molecular mechanism of drought response before/after WGD remains largely unclear.

**Result:**

We found that autoploid switchgrass (*Panicum virgatum* L.) 8X Alamo had higher drought tolerance than its parent amphidiploid 4X Alamo using physiological tests. RNA and microRNA sequencing at different time points during drought were then conducted on 8X Alamo and 4X Alamo switchgrass. The specific differentially expressed transcripts (DETs) that related to drought stress (DS) in 8X Alamo were enriched in ribonucleoside and ribonucleotide binding, while the drought-related DETs in 4X Alamo were enriched in structural molecule activity. Ploidy-related DETs were primarily associated with signal transduction mechanisms. Weighted gene co-expression network analysis (WGCNA) detected three significant DS-related modules, and their DETs were primarily enriched in biosynthesis process and photosynthesis. A total of 26 differentially expressed microRNAs (DEmiRs) were detected, and among them, sbi-microRNA 399b was only expressed in 8X Alamo. The targets of microRNAs that were responded to polyploidization and drought stress all contained cytochrome P450 and superoxide dismutase genes.

**Conclusions:**

This study explored the drought response of 8X and 4X Alamo switchgrass on both physiological and transcriptional levels, and provided experimental and sequencing data basis for a short-term adaptability study and drought-resistant biofuel plant breeding.

## Background

Switchgrass (*Panicum virgatum* L.), as a well-known bioenergy crop found on marginal lands [1], has a substantial potential to help relieve the world energy shortage [2]. It does not just have the dual purposes as a forage and energy crop [3], but it is even able to improve the aggregate stability of soil and its microbial biomass while reducing soil disturbance with a perennial rooting system [4]. Thus, since switchgrass is both an economical and environmentally friendly crop, its study and improvement merit substantial research. Recent studies have found that drought is a major limitation for biofuel production [5], and breeding objectives for switchgrass should address the enhancement of drought resistance.

Drought stress (DS) is now one of the most intractable global challenges. The latest report of World Meteorological Organization (WMO) indicates that large parts of Europe, Australia, Asia and South America experienced exceptional drought conditions owing to long-term warming trends, resulting in disruption of river transport and substantial agricultural losses (https://public.wmo.int/en/media/press-release/wmo-climate-statement-past-4-years-warmest-record). Previous research data shows that 3.2% of the global cereal production was lost owing to drought compared with an estimated counterfactual production over 2000–2007; moreover, the yield of cereal declined 4.9–5.2% with harvest area dropping by 4.0–4.3%, indicating that catastrophic drought can cause long-lasting crop failures [6]. With the increasing frequency of drought [7, 8] and slower recovery speed of global environment [9], the average crop loss rate reached 13.7% during more recent droughts [6]. Breeding objective to improve plant drought tolerance can be regarded as effective solutions to prevent more agricultural losses. Wide ranging studies on physiology, morphology and gene expression suggest that polyploidization can result in plants with increased amount of drought resistance [10–13].

Whole genome duplication (WGD) has occurred widely and has been studied in fungi [14, 15], bacteria [16], plants [17] and animals [18]. In plants, most spermatophytes arose through an ancestral WGD process followed by diploidization, and 70% of the current angiosperms are polyploids [19]. After the WGD process, the plants are able to adapt to environment changes more strongly [10, 20, 21, 22], owing to the triggering by a genomic rearrangement after rapid genomic shock [23]. For instance, after polyploidization, black locust (*Robinia pseudoacacia* L.) [24] and bulbous barley (*Hordeum bulbosum*) [25] became more resistant to salt. Moreover, the tetraploid Paulownia [12, 13] and Chenopodiaceae [10] showed higher drought tolerance than their diploid ancestors. Evidence showed that both autopolyploid (single chromosome set doubling in one species) and allopolyploid (multiple chromosome set forming by merging or doubling after hybridization) have a short-term adaptive potential that manifests through immediate changes in physiology and gene expression [20, 26, 27]. In Arabidopsis, an increase in the content of leaf potassium in natural tetraploid accessions contributes to their increased tolerance to salt [28]. The genes involved in stress response and hormonal regulation also have changes in expression in polyploids compared with their parental ploidies, such as in rice (*Oryza sativa*), sea barley (*Hordeum marinum*), and citrus (*Carrizo citrange*) [29–31, 21]. An enhanced understanding of plant short-term survivability after WGD is essential to address various challenges, such as climate change, water shortage, agricultural domestication [32] and natural adaption.

MicroRNA (miRNA) is commonly regarded as a type of crucial post-transcriptional regulator in plant stress responses. It is a type of single-strand endogenous noncoding RNA in length of approximately 22 nucleotides long that regulates gene expression by silencing mRNA via base pairing [33]. After the base pairing of miRNA and its target mRNA, the mRNA may be cleaved into two pieces, unstable with the shortened poly(A) tail, or inefficiently translated into proteins by the ribosome [34, 35]. Thus, the expression of target genes may be affected. Plant miRNAs are conserved in monocots and eudicots [36, 37], and their similarities are found in both mature miRNA and their coding genes. The conservation of plant miRNAs indicate that the expansion of plant miRNA gene families may have recently occurred and has not generated much divergence [38]. Studies clarify that some conserved miRNAs respond to drought in multiple type of plants, such as wheat (*Triticum aestivum* L.) [39], rice [40], Zhang, [41], tobacco (*Nicotiana tabacum* L.) [42], tomato (*Lycopersicon esculentum* Mill.) [43], cotton (*Gossypium* spp.) [44], sugarcane (*Saccharum officinarum*) [45], forage orchardgrass (*Dactylis glomerate* L.) [46] and the model plant Arabidopsis (*Arabidopsis thaliana* (L.) [47] as well. These studies indicate that miRNA156 can silence an SQUAMOSA-promoter binding like (SPL) gene to enhance the drought tolerance of alfalfa [48]. A miRNA166 knock-down rice line can survive drought stress by curling its leaves, reducing stomatal conductance and thinning its xylem vessels [41]. Finally, miRNA169 can reduce the degree of stomatal opening to decrease loss of water in leaves and control transpiration rate [43].

Expansion of the miRNA family could provide plants with altered gene expression, which could lead to changes in their adaptation to stress [49]. In addition, the expansion is largely due to WGD, and the number of miRNA genes increases at higher levels of ploidy [50]. After duplication, whether at the whole genome level or segmental, miRNA genes, similar to protein gene families, will randomly distribute and diversify in the current genome after expansion [51]. As a specific example, Liu find that tetraploid bulbous barley is more strongly adapted to salt stress than the diploid species, and this may be owing to a newly formed miR528b-3p in the tetraploid species [25]. However, the tetraploid bulbous barley is a natural autotetraploid species. Therefore, there might be some undiscovered differences in its genetic background between the tetraploid and diploid plants.

We sought to determine if WGD can result in stronger drought tolerance in switchgrass and observe the changes in gene expression at transcriptional level following WGD. Artificially doubled autopolyploid switchgrass (2*n* = 8*x* = 72) (8X Alamo) was generated by inducing its parental switchgrass cv. Alamo (2*n* = 4*x* = 36) (4X Alamo) by colchicine [30, 31]. In this study, we use 8X Alamo and 4X Alamo to process the drought treatment at various time points, followed by physiological and transcriptional analyses. Our previous study found that the sequence of 8X and 4X Alamo switchgrass were 99% identical with each other [52]. The analyses of this study could provide insights about drought the mechanism of response in switchgrass at both physiological and transcriptional levels before/after WGD and also provide abundant data for the drought tolerant breeding of switchgrass.

## Results

### Detection of drought tolerance of tetraploid and octoploid switchgrass

**Fig. 1:**
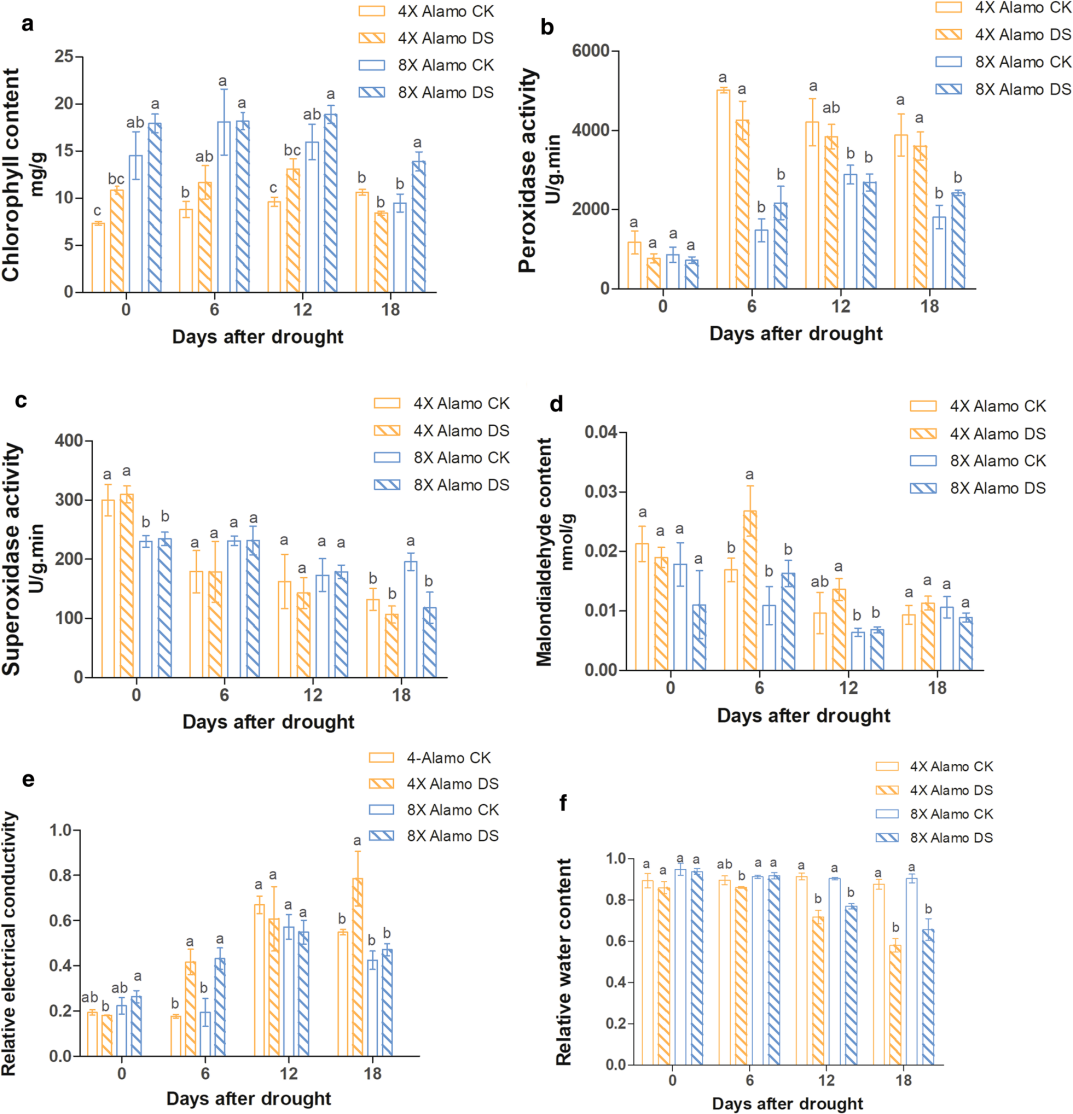
Physiological experiments of 8X Alamo and 4X Alamo switchgrasses. **a**–**f** physiology indicators under 0, 6th, 12th and 18th days of drought, orange columns refer to 4X Alamo, blue columns refer to 8X Alamo, hollow columns refer to control group, slashed columns refer to drought stress group. **a** Chlorophyll content. **b** Peroxidase activity. **c** Superoxide activity. **d** Malondialdehyde content. **e** Relative electrical conductivity. **f** Relative water content. Lower case letters a, b, c above the columns indicating the significance (*P* < 0.05) among different plant at same time point

Six drought resistant physiological indices were investigated under four different time points of drought stress by one-way ANOVA (SPSS 20.0) to detect the drought tolerance of autopolyploid switchgrass (8X Alamo) and its parental amphidiploid (4X Alamo) (Fig. 1). Chlorophyll can reflect plant drought tolerance as a positive indicator [53]. In this study, the content of chlorophyll in 8X Alamo was significantly (*P* < 0.05) higher than that in 4X Alamo, but they responded similarly to drought stress that increased at first and then was down regulated after day 12 of drought, while the chlorophyll content of stressed 4X Alamo dropped lower than that of control group, which exhibited the same trend previously identified in wheat [53] (Fig. 1a). Antioxidant enzymes, such as peroxidase (POD) and superoxide dismutase (SOD) can convert peroxides into less toxic or harmless substances during stress [54]. The POD activity in 8X Alamo was lower than that in 4X Alamo. In 4X Alamo, the POD activity was lower in drought stressed plants than in CK plants, but 8X Alamo had higher POD activity at 6 and 18 days in DS plant compared to CK plants. (Fig. 1b). The SOD activity in 4X Alamo was higher at the beginning, but it was reduced to a lower level than in 8X Alamo along plant growth and even significantly lower (*P* < 0.05) than 8X Alamo CK plant (Fig. 1c). Malondialdehyde (MDA) and relative electrical conductivity (REC) are two major indicators of membrane damage [55]. Changes in the content of MDA in both genotypes were similar in that they increased first and then decreased, but the MDA content in 4X Alamo was higher than in 8X Alamo indicating the increased permeability of plasma membrane in 4X Alamo (Fig. 1d). The REC increased in 4X Alamo in response to drought stress, while in 8X Alamo, the REC was increased initially responding to drought but then did not have much difference with CK plant. After day 12, the general REC of 8X Alamo was lower than 4X Alamo, and the REC of drought stressed 4X Alamo was significantly (*P* < 0.05) higher than CK plant (Fig. 1e). The relative water content (RWC) can reflect ability of leaf to retain water; thus, it is also an important indicator for the evaluation of drought tolerance [56]. In our study, the two ploidies did not differ greatly in their RWC, but the value was generally slightly higher in 8X Alamo (Fig. 1f). Collectively, the indicators described above showed that the switchgrass may have a stronger tolerance to drought stress following WGD.

### Analysis of RNA-sequencing for 8X and parental 4X Alamo switchgrass at different drought times

To identify the genes associated with WGD event and those that affected drought response, transcriptome libraries were established, which included 8X Alamo and 4X Alamo under five conditions (CK4, DS4_3, DS4_6, DS4_9, CK4_9, CK8, DS8_3, DS8_6, DS8_9, CK8_9; CK indicates control, DS indicates drought stress, and numbers after the dash indicates days of drought treatment) with three biological replicates. After high quality transcriptome sequencing from an Illumina Hi-Seq™ 4000 platform (Illumina, San Diego, CA, USA), we filtered the raw data, removed low-quality reads, and finally obtained 6.11–9.82 Gb clean reads of each library. Among all the samples, the GC content was more than 55.19%, while Q20 and Q30 were more than 96.12% and 90.95%, respectively (Additional file 1: Table S1), indicating that our sequencing data were high quality and reliable for further analysis. After mapping and aligning clean reads to the switchgrass reference genome (*P.virgatum* v4.1, Phytozome) using Bowtie v.2.2.3 and TopHat v.2.0.12., HTSeq v0.6.1 software was used to count the reads numbers mapped to each transcript to evaluate their expression. In total, 132,249 unique transcripts (fragments per kilobase of exon model per million reads mapped less than one, FPKM > 1) were obtained. To understand the effects produced by WGD and DS, seven comparisons (CK8vsCK4, CK8_9vsCK4_9, DS8_3vsDS4_3, DS8_6vsDS4_6, DS8_9vsDS4_9, CK4_9vsDS4_9, CK8_9vsDS8_9) were designed for DET (differentially expressed transcripts) analysis, after calculation by DESeq 2 (|log2FoldChange| > 1, *P* < 0.05) we obtained a total of 36,109 unique DETs totally (Additional file 2: Table S2).

#### Annotation of drought-related DETs Showed a difference before/after WGD

**Fig. 2:**
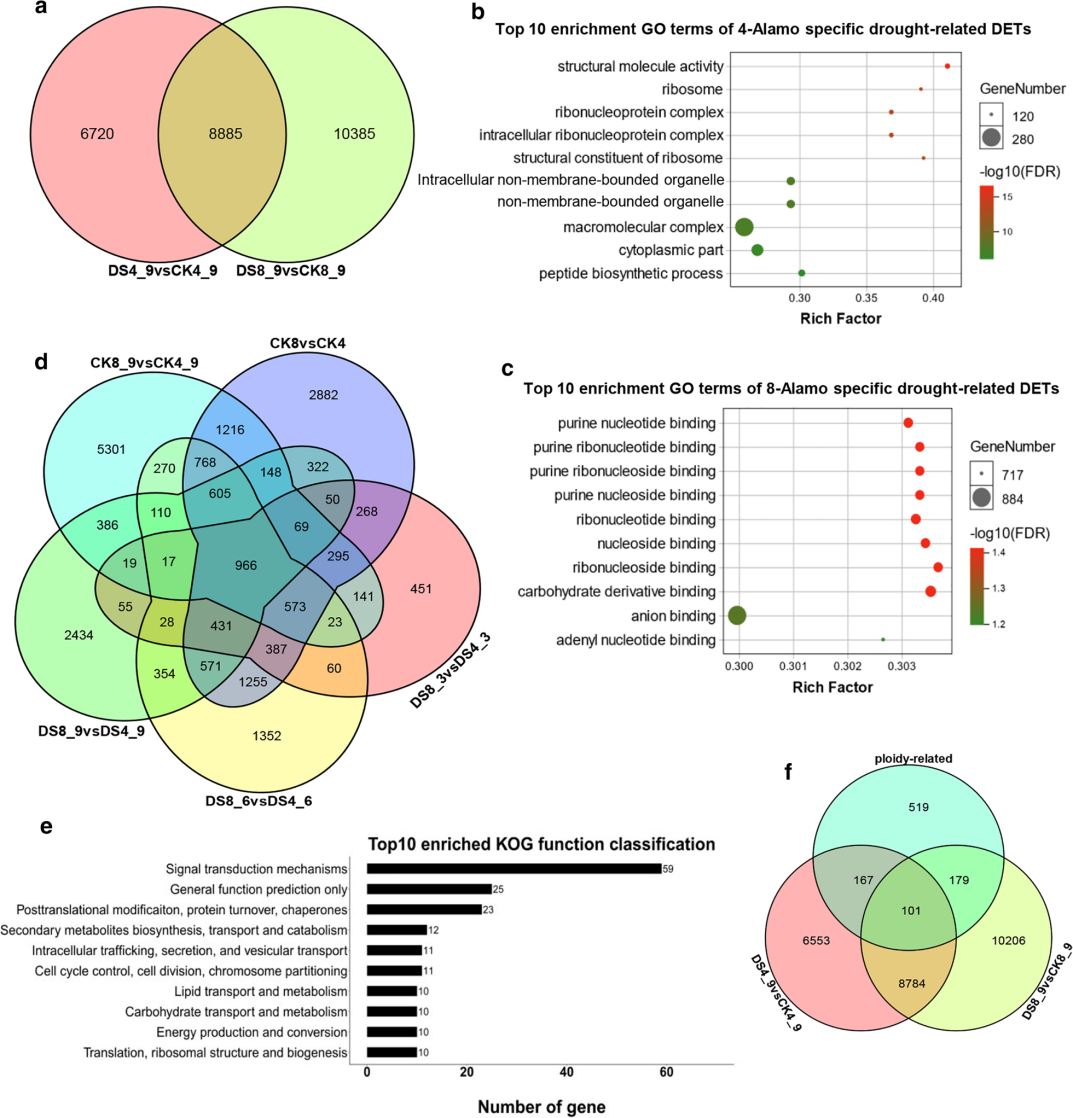
Differently expressed transcripts analysis. **a** Venn gram of drought-related DETs in two ploidies; **b** Top 10 enrichment GO terms of 4-Alamo specific drought-related DETs; **c** Top 10 enrichment GO terms of 8-Alamo specific drought-related DETs; **d** Venn gram of ploidy-related DETs; **e** KOG functional enrichment of 966 shared ploidy-related DETs; **f** Venn gram of drought-and ploidy-related DETs in two ploidies

To determine the difference in drought response between 8X Alamo and 4X Alamo, we compared the DETs from DS8_9vsDS4_9 and CK4_9vsDS4_9 (Fig. 2a). There were 6720 and 10,385 specific drought-related DETs in 4X Alamo and 8X Alamo, respectively, and they shared 8885 common DETs that responded to drought stress. After GO analysis, the most enriched GO terms for specific DETs in 4X Alamo focused on structural molecule activity, ribosome, macromolecular complex and peptide biosynthesis process (Fig. 2b). In 8X Alamo, the most enriched functions were related to purine nucleotide, nucleoside and carbohydrate derivative binding (Fig. 2c).

For ploidy-related DETs, the intersection of five comparisons was defined which included 966 DETs (Fig. 2d). A pathway analysis of the 966 ploidy-related DETs showed that 8X Alamo and 4X Alamo may differ in their mechanism of signal transduction pathways; in addition, these DETs that were most significantly enriched in the GO term ADP binding (FDR < 0.001) and KEGG enrichment also showed that those DETs were associated with Vitamin B6 metabolism and phosphonate (*P* < 0.01) (Fig. 2e, Additional file 3: Fig. S1).Among these transcripts, there were 167 and 179 DETs that were also found to be specifically drought-related in 4X Alamo and 8X Alamo, respectively, and they were significantly (FDR < 0.001) enriched in macromolecular complex-related functions and ATP synthase-related functions, respectively, which indicated the different drought response mechanism caused by WGD (Fig. 2f, Additional file 4: Fig. S2).

#### DETs that responded to drought and/or WGD through WGCNA

**Fig. 3:**
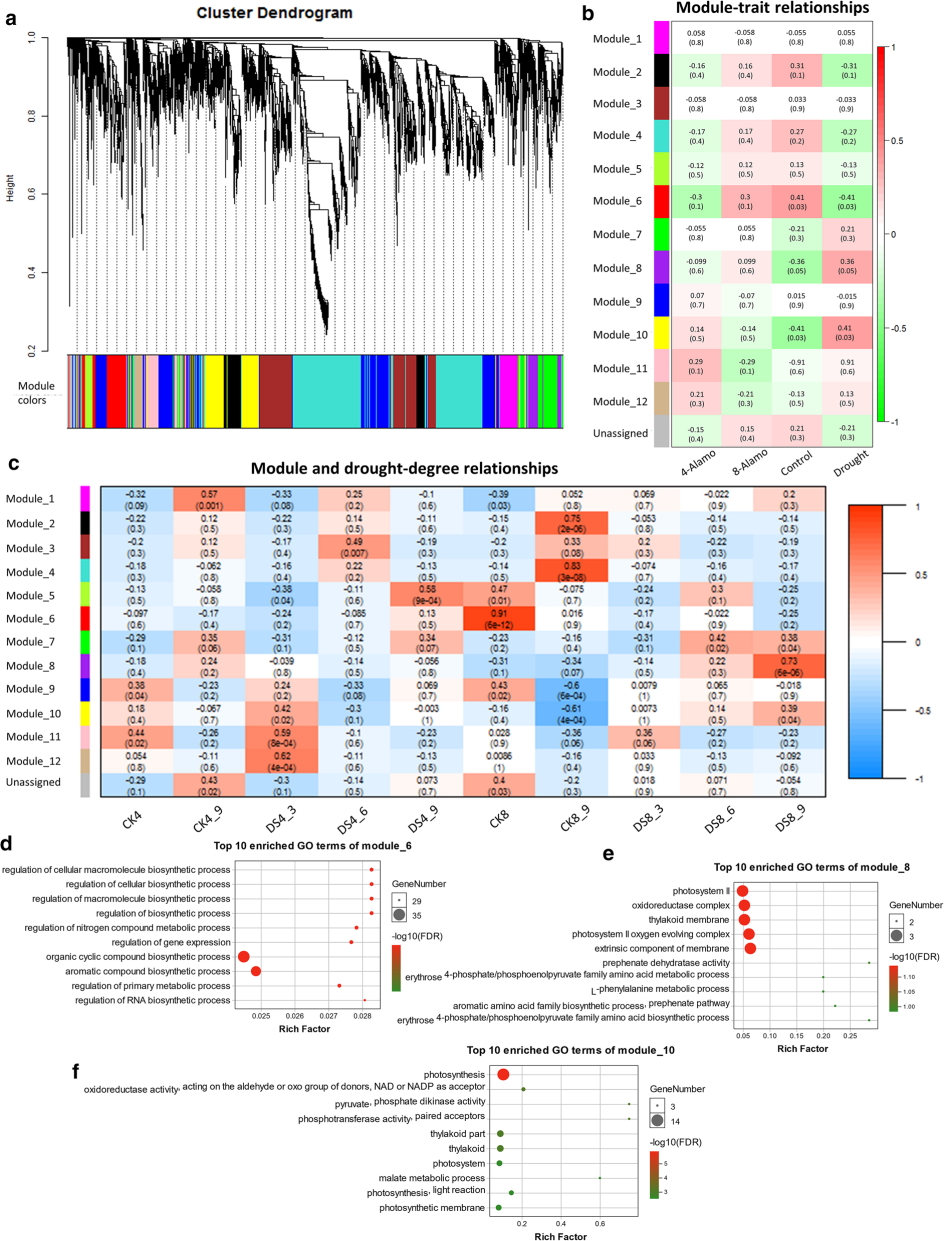
Weighted gene co-expression network analysis for DETs. **a** cluster dendrogram; **b** Module-trait relationships with ploidy and drought traits; **c** Module and drought-degree relationships; **d** Top 10 enrichment GO terms of Module_6; **e** Top 10 enrichment GO terms of Module_8; **f** Top 10 enrichment GO terms of Module_10. The numbers in **b** and **c** indicates the correlation coefficient (top) and significance P-value (bottom) between modules and traits

Owing to the multiple time points, WGCNA was conducted for the 10 sets of conditions (CK4, CK4_9, DS4_3, DS4_6, DS4_9, CK8, CK8_9, DS8_3, DS8_6, DS8_9). After removing the low expression DETs (the maximum FPKM in 10 conditions was lower than five) [57] from the seven comparisons described above, 23,262 DETs were inputted for analysis. According to the correlation with drought stress and ploidy, we obtained 12 transcript expression modules, including 5611 DETs in the range of 107–1621 DETs per module (Fig. 3a).

Module_6 showed a significant correlation with the control (positive) and drought (negative) trait, and also had a stronger correlation with the two ploidies than other modules (Fig. 3b). In addition, it showed a noticeable correlation with CK8, and the GO enrichment showed that the DETs of module_6 functioned in the regulation of cellular macromolecular biosynthetic process (Fig. 3c, d). There were two other modules that had a significant positive correlation to drought stress: module_8 and module_10, but they had opposite correlation to ploidies (Fig. 3c). Module_8, which had positive relation with drought stress and 8X Alamo, had significantly (FDR < 0.001) enriched GO functions, such as photosystem II, oxidoreductase complex and thylakoid membrane (Fig. 3e). Module_10 had a positive relation with 4X Alamo and was enriched in photosynthesis, oxidoreductase activity, pyruvate phosphate dikinase activity and photosynthesis light reaction (Fig. 3e).

### MicroRNA sequencing result and target analysis

#### MicroRNA sequencing of tetraploid and octoploid switchgrass under drought stress

Three timepoints with six sample groups (CK4, DS4_3, DS4_6, CK8, DS8_3, DS8_6) were subjected to miRNA sequencing. They ranged from 0.612 Gb to 0.747 Gb; the sizes of data of two ploidies did not differ significantly, but all were high quality (Additional file 5: Table S3). According to the mapping result of reads to reference and miRbase, 69 conserved miRNAs were detected without the identification of any novel miRNAs.

**Fig. 4:**
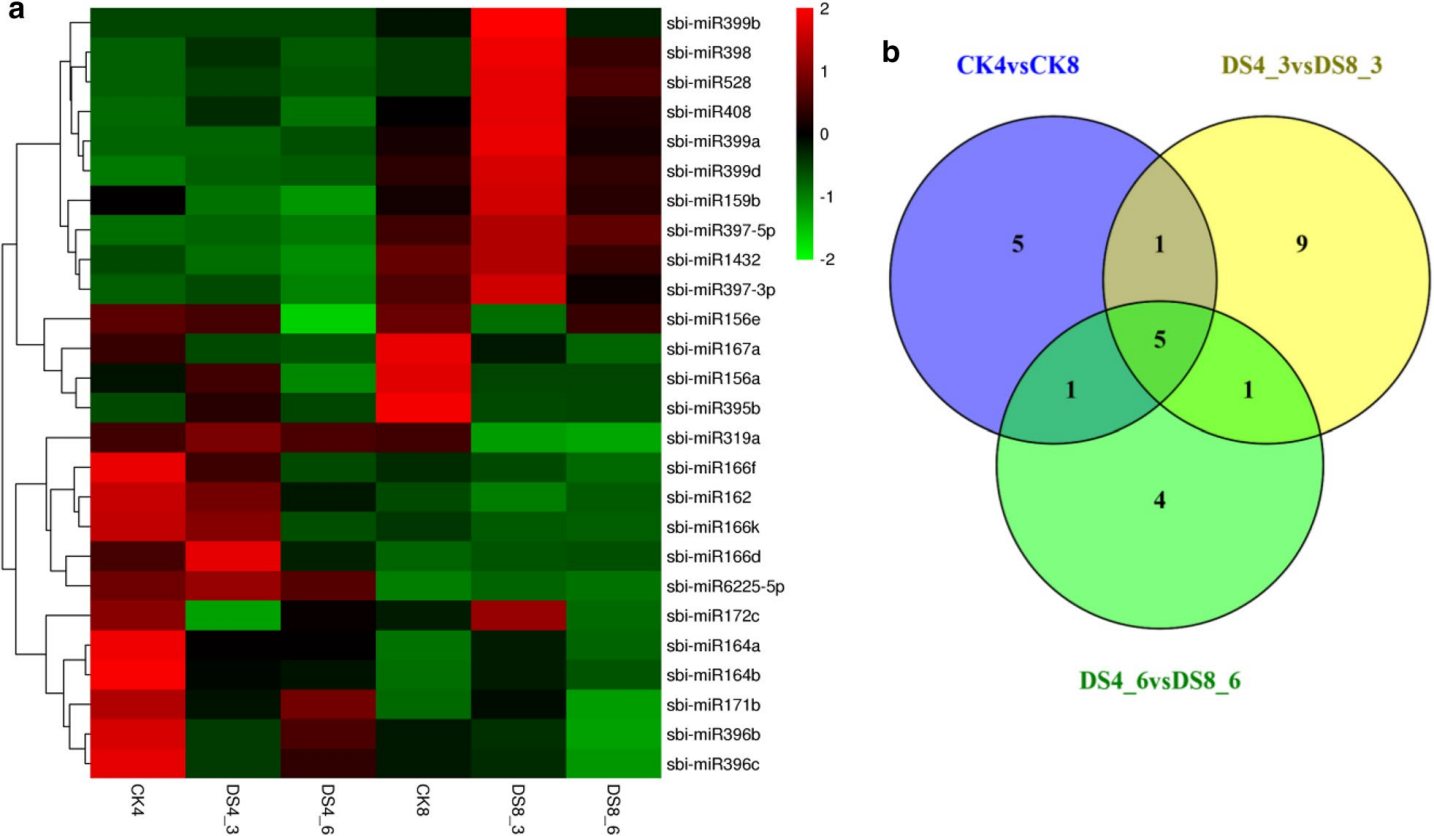
Differently expressed miRNAs. **a** Cluster analysis of 33 differently expressed miRNA. Each cell represents the expression of microRNAs at every treated time point. The expression data of microRNAs are standardized by Z-Score, ranging from − 2 to 2, and the color ranges from green to red. **b** Venn diagram for ploidy comparisons

After the calculation of DESeq 2, a total of 26 differentially expressed miRNAs (DEmiRs) were generated from three comparisons (CK8vsCK4, DS8_3vsDS4_3, DS8_6vsDS4_6). The three replicates of each sample were averaged to process normalization of expression of DEmiRs, which produced readcount-TPM data (Table 1). Among these normalized data, sbi-miRNA399b was not expressed in 4X Alamo, but it was expressed in 8X Alamo. After combining the expression data and cluster heatmap, sbi-miRNA6225-5p had a significant higher level of expression in 4X Alamo (Table 1, Fig. 4). From the comparison between parental 4X and autoploid 8X Alamo (CK8 vs CK4; DS8_3 vs DS4_3; DS8_6 vs DS4_6), five miRNAs (sbi-miR397-5p, sbi-miR408, sbi-miR528, sbi-miR399d, sbi-miR397-3p) were found to be differentially expressed at three timepoints and they were clustered closely; thus, we considered them to be WGD-related miRNAs (Fig. 4). In addition, sbi-miR399b, which was specifically expressed after WGD, had a similar trend of expression with these five miRNAs (Fig. 4a). Since we had obtained data indicating that 8X Alamo may have stronger drought tolerance, these miRNAs might be important regulators that were affected by WGD and then functioned on drought adaptation.

**Table 1:** Expression data of the 26 differently expressed miRNAs in all sample groups

DE-miRNAs	CK4.tpm	DS4_3.tpm	DS4_6.tpm	CK8.tpm	DS8_3.tpm	DS8_6.tpm
sbi-miR1432	1478.357	1008.258	609.6439	3745.842	4724.229	3131.075
sbi-miR156a	1528.248	1749.053	1233.591	2147.256	1405.639	1407.752
sbi-miR156e	2619.888	2561.149	1652.226	2669.33	1975.242	2508.514
sbi-miR159b	321.938	181.7307	140.8163	342.6949	569.9083	363.1975
sbi-miR162	28,704.81	24,097.3	16,614.4	13,922.78	11,035.56	12,906.97
sbi-miR164a	1824.652	968.8054	962.4328	519.1354	845.7595	582.7609
sbi-miR164b	556.258	259.1397	248.0747	138.9058	232.4262	169.6744
sbi-miR166d	117,270.3	156,333.6	93,382.52	77,060.11	79,944.65	81,853.8
sbi-miR166f	7420.48	5327.581	3804.693	4166.297	3776.898	3427.617
sbi-miR166k	8702.091	7920.625	5138.521	5474.473	5028.839	4952.296
sbi-miR167a	6632.837	4208.917	3991.304	10,076.97	5225.41	3720.673
sbi-miR171b	679.8532	452.4544	609.313	344.5755	458.0563	283.1764
sbi-miR172c	616.6473	196.4381	434.0128	385.1808	636.6969	275.6394
sbi-miR319a	227.1437	258.7373	234.9128	228.8699	106.699	102.6096
sbi-miR395b	44.14,665	382.6676	54.75367	996.3083	43.11919	56.47531
sbi-miR396b	5662.458	2535.809	4062.957	2959.91	2662.763	1337.399
sbi-miR396c	5347.586	2049.269	3264.826	2461.489	2215.095	993.4223
sbi-miR397-3p	336.5019	397.0556	250.4491	792.8943	1115.126	618.9932
sbi-miR397-5p	4290.472	6760.029	2355.667	46,830.25	75,060.34	54,178.83
sbi-miR398	5.289158	183.7408	34.86915	136.694	1219.584	553.0493
sbi-miR399a	0	0	4.35286	24.66964	65.54629	24.11193
sbi-miR399b	0	0	0	34.85254	216.0373	23.89048
sbi-miR399d	225.2285	363.5057	398.8155	1130.597	2048.343	1152.142
sbi-miR408	581.0438	1772.698	342.7141	2742.683	7251.326	3352.049
sbi-miR528	190.2462	1226.98	390.5228	1423.397	11859.53	6234.067
sbi-miR6225-5p	44.49316	51.22464	39.6321	1.99408	6.47738	4.332234

Since miRNAs primarily function by regulating the expression of their targets, we predicted the targets of the 26 DEmiRs using the online software psRNATarget. A total of 615 transcripts were predicted, while 445 of them were DETs of the RNA-Seq result, and the target transcripts of each DEmiR ranged from 3 to 52 (Additional file 6: Table S4).

#### Integrated analysis of ploidy-related microRNAs and their predicted targets

Based on the analysis above, five ploidy-related miRNAs were found, and their trends of expression were similar. Their level of expression was significantly lower (*P* < 0.05) in 4X Alamo than in 8X Alamo.

The expression cluster analysis (Additional file 7: Fig. S3) showed that a large proportion of sbi-miR399d targets responded to DS. Among these targets, some of them, which were annotated to metabolism process, were only upregulated at the late drought period in 8X Alamo. While sbi-miR399d showed upregulation first and followed with a decrease, the expression changes of these “metabolism process” targets did not occur simultaneously with sbi-miR399d (Fig. 4). The same trend was found in part of the sbi-miR397-3p and sbi-miR397-5p targets. Moreover, the targets that were annotated to cell and cell part changed dramatically at late DS period in 8X Alamo. More than half of the sbi-miR528 targets had different trends of expression before/after WGD.

Sbi-miRNA399b was specifically expressed in 8X Alamo, and the ploidy-related miRNA sbi-miRNA528 was closely clustered with it, as well as sbi-miRNA398. Since 8X Alamo was found to have stronger drought tolerance, these three miRNAs were analyzed to detect their role in drought response after WGD.

**Fig. 5:**
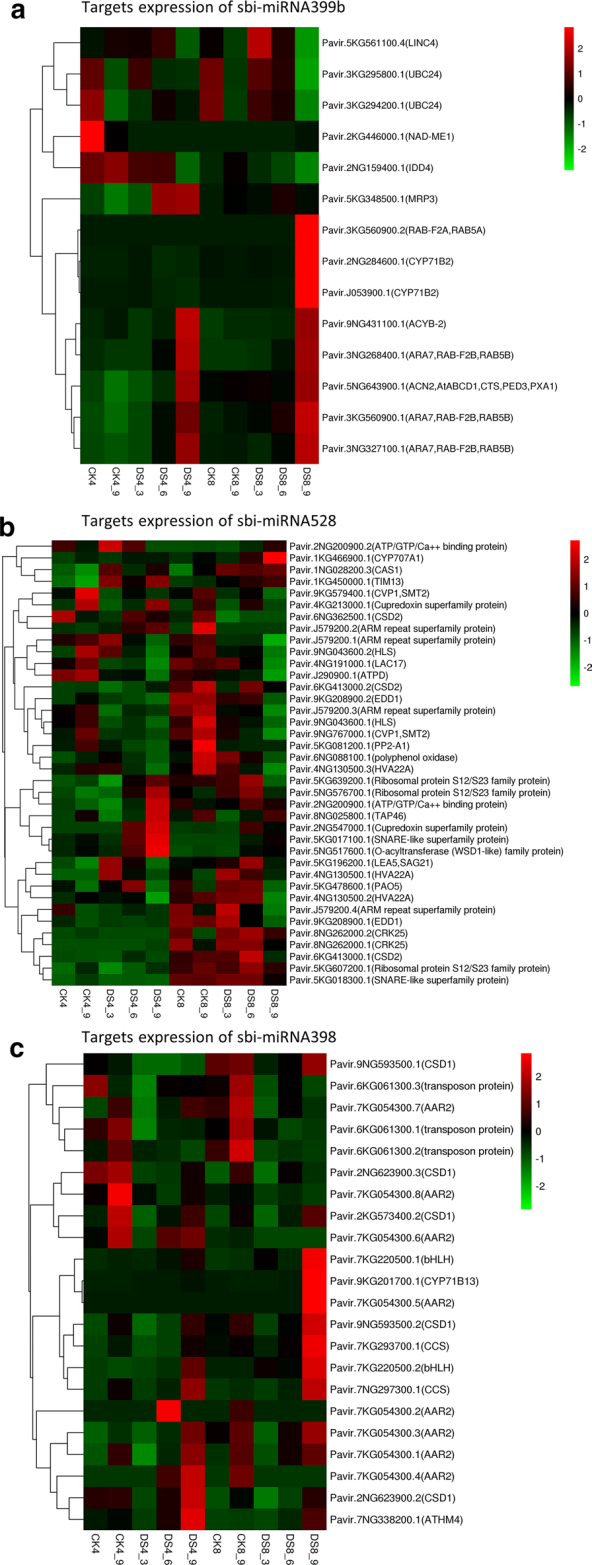
Targets expression analysis of three ploidy- and drought- related miRNAs. **a** Expression analysis of sbi-miRNA399b targets; **b** Expression analysis of sbi-miRNA528 targets; **c** Expression analysis of sbi-miRNA398 targets

From the cluster heatmap of sbi-miRNA399b targets (Fig. 5a), we found several targets had a different pattern of drought response before and after WGD: Pavir.2KG446000.1 (NAD-ME1), Pavir.2NG159400.1 (IDD4), Pavir.5KG348500.1 (MRP3), Pavir.3KG560900.2 (RAB-F2A,RAB5A), Pavir.2NG284600.1 (CYP71B2), Pavir.J053900.1 (CYP71B2). Among them, Pavir.5KG348500.1, a resistance-associated protein 3, was upregulated with drought stress in 4X Alamo but did not show any obvious changes in 8X Alamo. Two cytochrome P450 targets (CYP71B2) Pavir.2NG284600.1 and Pavir.J053900.1 were highly expressed during the late drought period in 8X Alamo. These levels of expression could have been related to the decrease in expression of sbi-miRNA399b at the late drought period based on miRNA regulation pattern.

Sbi-miRNA528 and sbi-miRNA398 were clustered together with sbi-miRNA399b. They were significantly (P < 0.05) differentially expressed in 4X Alamo and 8X Alamo. In addition, they were detected to be DEmiRs in 8X Alamo. In 4X Alamo, they were expressed at low levels without obvious changes during drought, but in 8X Alamo, their levels of expression increased with drought. In 8X Alamo, they were slightly down regulated at the sixth day of drought. Owing to pattern of miRNA regulation, they could play important roles in the resistance of 8X Alamo to drought; thus, their key targets were analyzed (Fig. 5b, c).

Pavir.J290900.1 was a target of sbi-miRNA528 and annotated as an ATP synthase delta-subunit gene. This target had a pattern of downregulation that responded to drought, and its level of expression was lower after WGD. In addition, there was an ATP/GTP/Ca++ binding protein gene Pavir.2NG200900 among the targets of sbi-miRNA528, these genes have a lower level of expression after WGD and were upregulated with drought but down-regulated in 4X Alamo. Alternatively, some targets, such as cysteine-rich receptor-like protein kinase 25 (CRK25) and copper/zinc superoxide dismutase 2 (CSD2), had a significantly (*P* < 0.05) higher level of expression in 8X Alamo. Similarly, to sbi-miRNA399b, sbi-miRNA528 also had a cytochrome P450 target (CYP707A1) which was significantly (P < 0.05) up-regulated at the late drought period.

Among the targets of sbi-miRNA398, the CSD1s reacted in same manner with CDS2s targeted by sbi-miRNA528 and had a higher level of expression after WGD and were upregulated with drought. From Fig. 5b, several targets (from bHLH to CCS) were significantly (*P* < 0.05) upregulated at the late period of drought in 8X Alamo, and among them there was one cytochrome P450 target Pavir.9KG201700.1 (CYP71B13) and one copper/zinc superoxide dismutase 1 Pavir.9NG593500.2 (CSD1).

In summary, the cytochrome P450 targets were found in the targets of all three miRNAs and these CYP targets shared the same trend towards WGD and drought stress. CSD genes were found in both the sbi-miRNA528 and sbi-miRNA398 targets.

## Discussion

Short-term survivability after WGD results in a greater degree of stress resistance in the plant in most cases [23]. Thus, studies that focus on the expression of changes in key genes are deemed to be essential for further mechanistic research and biofuel plant breeding. In our study, we first investigated the drought tolerance of both tetraploid and octoploid switchgrasses at physiological level and analyzed their microRNA and gene expression of at molecular level to try to determine the impact of WGD through combination analysis and determine if it is an efficient way to improve drought tolerance of biofuel plant.

### Stronger drought tolerance of 8X Alamo

When plant suffer from a water deficit, their chlorophyll content decreases over long-term stress; antioxidant enzyme defense systems are weakened, and lipid peroxidation is enhanced in the leaves [53]. We found that 8X Alamo had higher chlorophyll and SOD contents than 4X Alamo during DS indicating that 8X Alamo might have a mechanism of protection that is more effective against oxidative damage under DS. The content of chlorophyll reflects drought tolerance of plant to some degree [53], and SOD can eliminate toxic or harmful substances [54]. The chlorophyll content was higher in drought stressed 8X Alamo than that in CK at days 0, 12 and 18 (significant, *P* < 0.05) (Fig. 1a), this might because of the individual difference of drought and untreated plants, and this phenomenon need to be further investigated. Although the activity of antioxidant enzyme POD was lower in 8X Alamo than in 4X Alamo, it gradually increased in both ploidies as they responded to DS, which is consistent with the studies of DS on other plants [58]. This increase suggests the ability to maintain induced activity of antioxidant enzymes in the two types of switchgrass. The lipid peroxidation rate, which indicates the degree of membrane damage, is assessed by measuring the dominant product MDA and REC, and an increase in the amounts of MDA and REC indicate that the plants have suffered severe damage owing to DS [ 55, 59]. In our study, the membrane damage indicators MDA and REC were generally lower in 8X Alamo during drought, indicating that the 8X Alamo switchgrass had a stronger tolerance than 4X Alamo. In addition, a high RWC in plant leaves reveals the higher resistance under DS, and high RWC is the result of increased osmotic regulation [60]. In our study, the RWC of 8X Alamo and 4X Alamo did not have significant difference, but it was slightly higher in 8X Alamo at each drought time point. Thus, our results could present the higher drought tolerance of 8X Alamo.

### Transcriptome comparison of tetraploid and octoploid switchgrass in response to drought

To compare the biological functions of DETs in two ploidies that responded to drought stress, annotation was processed for the DETs of two ploidies. 4X Alamo-specific drought-related DETs (Fig. 2a, b) were enriched in structural molecule activity, macromolecular complex, peptide biosynthesis process and cytoplasmic part, which indicated that they may relate to the structural response mechanism of membrane. When the plants are subjected to DS, the open conformation loop D of aquaporin is displaced, and this movement opens a hydrophobic gate that can block the entrance from the cytoplasm; this molecular gating mechanism is conserved in all plant plasma membrane aquaporins [61], thus the drought-related DETs might been associated in this structure conformation process. Moreover, in our study, the drought-related DETs in 4X Alamo were significantly enriched in ribosome-related functions (FDR < 0.01) (Fig. 2b). From reported studies, when facing stress, ribosome inactive proteins could be induced and contributes to defense mechanisms [62]. More than 50% of the ribosomal protein genes were upregulated in shoot and root tissues of rice, they might have a common role in inducing tolerance under drought stress [63].

The enriched binding activities of nucleoside and nucleotide in 8X Alamo might be the main difference with 4X Alamo when facing DS (Fig. 2c). As a parallel with drought-related DETs specifically in 8X Alamo, in rice, some phosphoproteins, including nucleotide-binding protein, ribonuclease and ribosomal protein have been identified as drought-responsive proteins [64].

Ploidy-related DETs were enriched in signal transduction mechanism pathways, (Fig. 2e). A previous study showed that genome duplication improves signal transduction resulting in the enhanced perception of environmental signals [65], and this could explain the higher drought tolerance of 8X Alamo in our study. Moreover, among these ploidy-related transcripts, 179 DETs were found to be specifically drought-related in 8X Alamo, and they were significantly enriched (FDR < 0.001) in ATP synthase-related functions (Fig. 2f, Additional file 4: Fig. S2).

Twelve gene expression modules were detected by WGCNA analysis for the bulk transcriptome data. Three modules were found to have a significant correlation with DS (negative: module_6; positive: module_8 and module_10). The genes in module_8 and module_10 were primarily concentrated in photosynthesis-related pathways, oxidoreductase complex and phosphorylation activity, which were consistent with previous studies. Drought stress has direct (stomatal limitation) and indirect (oxidative stress caused by multiple stress overlap) effects on photosynthesis [66]. According to our physiological experiment, the chlorophyll content of 8X Alamo was significantly (*P* < 0.05) higher than that of 4X Alamo, and the chlorophyll content positively correlated with photosynthetic rate. Therefore, it is highly likely that this was the reason for the difference in drought resistance between 4X and 8X Alamo. Chloroplasts are the main sites of photosynthesis. After the chloroplasts have been exposed to drought stress, they can regulate changes in gene expression, ion transport on plasma and vacuole membranes through retrograde ion signals and ion transport, thereby controlling root elongation, stomatal opening and closing, waxy layer formation and the osmotic pressure balance [12, 13].

#### Gene expression of retrograde signal pathway in the chloroplast

**Fig. 6:**
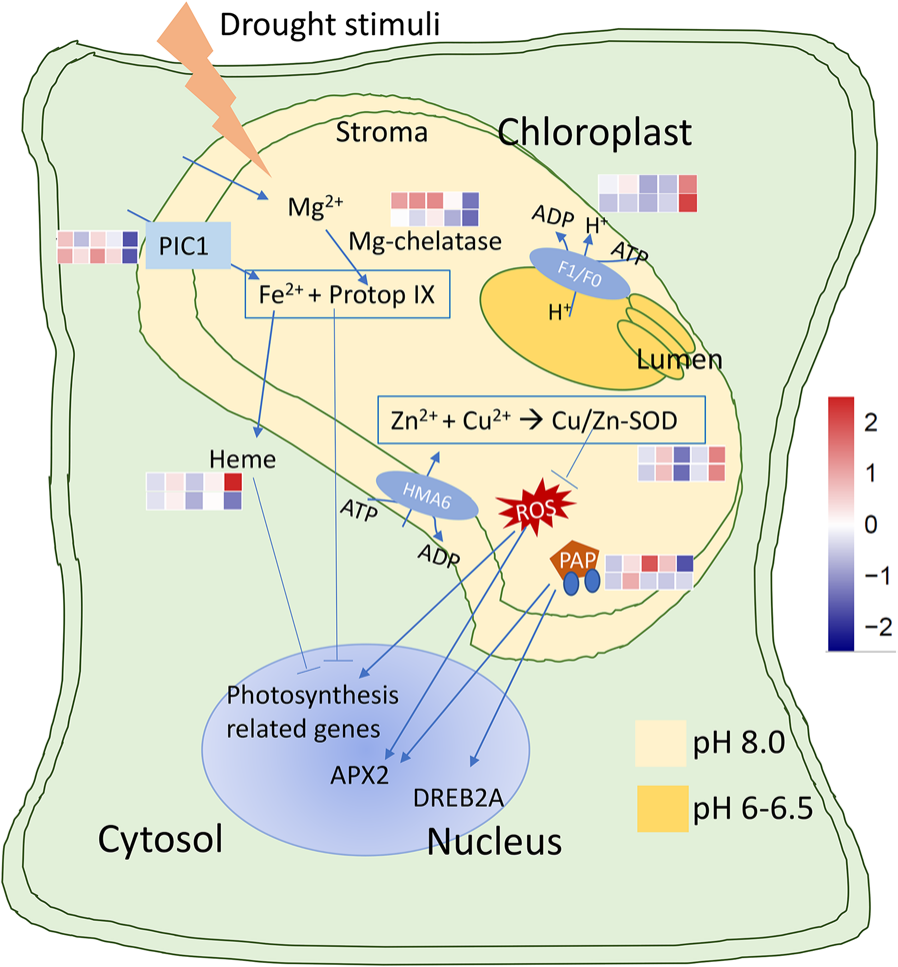
Retrograde signal pathways of plant chloroplast. Arrow represents positive regulation, T arrow represents negative regulation; Cubes represent to expression level in CK4, CK4_9, DS4_3, DS4_6, DS4_9 (top row), CK8, CK8_9, DS8_3, DS8_6, DS8_9 (bottom row) from left to right, respectively. The FPKM value of the included genes were normalized together using Z-score by online platform OmicShare (http://www.omicshare.com/tools). Red color means highest value, while blue color means the lowest

The most enriched DETs in module_8 and module_10 (both related to DS) were related to photosynthesis; thus, changes in gene expression in the chloroplast signal pathway [12, 13] were analyzed in this study (Fig. 6). Mg chelatase was associated with negative regulation to photosynthetic-related genes in the nucleus [67]. Thus, its lower expression in 8X Alamo could have contributed to the higher expression of photosynthetic genes in these types of plants. In addition, the heme downstream of Mg chelatase significantly (*P* < 0.05) increased between the sixth and ninth days of drought in parental switchgrass while simultaneously significantly (*P* < 0.05) decreasing in 8X Alamo. Heme can suppress photosynthetic-related gene expression in the nucleus [68], [69]. Therefore, a higher abundance of the expression of photosynthetic-related genes could appear after WGD. Another signal component 3′-phosphoadenosine 5′-phosphate (PAP) which transports signals from the chloroplast to nucleus, was upregulated followed by down regulation when it responded to drought in 4X Alamo. PAP can positively regulate the expression of ascorbate peroxidase (APX2) and DREB2A transcription factors which are key regulators of plant drought responses [70, 71]. However, in 8X Alamo it did not show any obvious changes but had was expressed at a higher level than that in 4X Alamo at a late period of drought. The ATP synthase gene had a sudden increase in expression between the sixth and ninth days of drought, and it was more significant after WGD, which could provide a greater supply of energy to the autoploid to counteract drought stress. Copper/zinc superoxide dismutase (CSD) genes had a similar trend of expression in the two ploidies, and they were all expressed at higher levels in response to drought, which reflected the fact that CSDs are drought response genes. This finding is consistent with that in peanut [72].

### MicroRNAs that induced by duplication and also related to drought response

MicroRNA sequencing identified a member of the miRNA399 family that is only expressed in 8X Alamo. Its sequence was aligned to sbi-miRNA399b of sorghum. Under drought stress, the expression of sbi-miRNA399b first increased and then was followed by a decrease. The miRNA399 family is the first low phosphorus stress responsive microRNA family detected in plants, and the expression of miRNA399 in maize is negatively correlated with the ability to resist low-phosphorus stress [73]. Studies in Arabidopsis found that miRNA399b was sensitive to environmental temperature. Overexpressed miRNA399b in Arabidopsis at normal temperature (23 °C) resulted in early flowering [74]. Changes in the expression of microRNA399 family under abiotic stress in plants revealed that microRNA399d could promote plant growth under abiotic stress conditions [75]. Arabidopsis in which miRNA399b was overexpressed was more tolerant to salt stress and exogenous ABA but was sensitive to drought [76]. In our study, 8X Alamo was more drought tolerant, but it remains to be proved whether this tolerance was directly affected by the expression of sbi-miRNA399b.

Two microRNAs, sbi-micro528 and sbi-micro399b, which were the most similar to expression of sbi-microNA399b, were differentially expressed in response to ploidy changes and increased first and then decreased slightly in 8X Alamo with the drought time. However, in a study of wheat, both miR528 and miR398 responded to drought stress but had a different pattern of regulation: miR528 was downregulated, and miR398 was upregulated [77]. MiR528 has been shown to be positively correlated with the accumulation of ROS [78], [79] and negatively regulates resistance to disease in rice [79]. MiR398 is directly related to the plant stress regulation network. It regulates plant responses to copper and phosphorus deficiency and oxidative, drought, salt, ultraviolet radiation and other stresses. The up-regulation of miR398 in alfalfa [80] and wheat [77] was detected under water stress, which was consistent with our results. Compared with other plant studies, the different drought response pattern of microRNA528 in 8X Alamo and its specific role merit further study.

### 8X Alamo specific microRNAs and their target genes

**Fig. 7:**
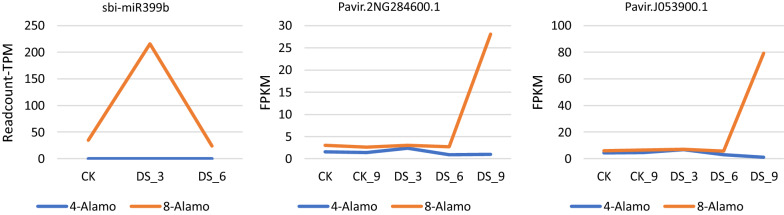
Expression of sbi-miRNA399b and CYP71B2 under different drought time

Sbi-miRNA399b was only found in 8X Alamo. Its target Pavir.5KG348500.1 is a resistance-associated protein 3, and upregulated with drought stress in 4X Alamo but did not change in 8X Alamo, indicating that sbi-miRNA399b might be involved in repressing the expression of Pavir.5KG348500.1. On the contrary, two CYP71B2 genes, Pavir.2NG284600.1 and Pavir.J053900.1, were also identified by target gene prediction, and the level of their expression is very low in 4X Alamo (Fig. 7). In 8X Alamo, the expression of sbi-miRNA399b was up-regulated in the early stage of drought, while the expression of the two targets was relatively low. When the miRNA was down regulated during the late stage of drought, the level of expression of two targeted CYP71B2 genes were up-regulated, suggesting that sbi-miRNA399b might negatively regulate the two CYP71B2 genes.

CYP71B2 is designated cytochrome P450, 71 family, subfamily B, polypeptide 2. Many members in Cytochrome P450 subfamily has been proven to be involved in the regulation of ABA in sweet cherries, which can delay fruit ripening and regulate the content of ABA during fruit development to manage water stress [81]. In addition, CYP707A3 in Arabidopsis can balance the threshold level of ABA during drought and rehydration [82]. Among the proteins of cytochrome P450 family 71 that have been studied, these proteins are mostly involved in the regulation of sesquiterpene lactone metabolism [83], [84]. Further research is merited to identify the specific roles of these two CYP71B2s that we identified.

## Conclusion

The samples of 4X and 8X Alamo switchgrass were collected after drought treatment. The physiological results showed that 8X Almao switchgrass had stronger resistance to drought.

In particular, drought-related DETs specifically in 4X Alamo were enriched in structural molecule activity, while in 8X Alamo, they were enriched in purine nucleotide binding. When considering the influence of both drought and WGD, 167 transcripts were detected in 4X Alamo and most were significantly enriched (FDR < 0.001) in the structural molecule activity. A total of 179 transcripts in 8X Alamo were most significantly enriched (FDR < 0.001) in mitochondrial proton-transporting ATP synthase complex, indicating the difference in main mechanistic responses towards DS. The results of WGCNA also showed that the drought response module was primarily annotated in photosynthesis whether it is positively related to WGD or not.

A total of 69 conservative microRNAs were obtained with 26 of them differentially expressed, and no novel miRNA was found. Among them, sbi-miRNA399b was only expressed in 8X Alamo. It was predicted that its target gene cytochrome P450 (CYP71B2) is related to the regulation of ABA. The target genes of microRNAs in response to ploidy variation were annotated in biological functions such as electron transfer activity, magnesium ion transporter, ubiquitinase, SnRK2s, CSD2s, and catalytic activity. Ploidy-related miRNA sbi-miRNA528 and sbi-miRNA398 were clustered closely to sbi-miRNA399b. Their target genes were primarily CSD2 and CYP71B2 genes, and they were significantly (P < 0.05) up-regulated in the late stage of drought resistance in 8X Alamo.

The higher content of chlorophyll and the low expression of heme and magnesium chelatase in 8X Alamo might contribute to the expression of photosynthesis-related genes in nucleus and then improve drought tolerance after WGD.

## Methods

### Plant materials and treatments for physiological experiment

#### Plant materials

In this study, 4X Alamo and 8X Alamo switchgrass were grown in pots and taken in the greenhouse (Wenjiang, Sichuan, China) at 28 °C/20 °C (day/night) with a photoperiod of 16 h/8 h (day/night), using mixed soil in each pots (peat moss: vermiculite: perlite = 1:1:1). Each ploidy had six biological repeats (six pots). Three pots were treated as control group and three pots were treated with drought when they all grew to 4–5 leaves. Under natural drought stress, water was irrigated to saturation before stress, so that each pot material maintained 80% of soil moisture. Leaf samples in the middle part of seedlings were collected at 6, 12 and 18 days after stress, and the index changes of these seedlings at different time points were determined to analyze and compare drought resistance of two ploidy switchgrass germplasms.

#### Measurement of indicators

Chlorophyll content was measured according to the procedures given by Arnon [85]. Relative water content of second leaf for each sample was measured by weighing method [86]; Relative electrical conductivity was measured by conductometer method [87]; Malondialdehyde (MDA) was determined by thiobarbituric acid method [88]; Peroxidase (POD) activity was measured by Britton and Mehly’s method [88]. Superoxide (SOD) activity was determined using Riboflavin-NBT method [89]. The significance was calculated using one-way ANOVA (SPSS 20.0). The specific protocols were described in Additional file 8.

### Plant materials and treatments for sequencing

#### Plant materials

For sequencing, the same materials of physiological experiment were used. One plant of each ploidy type was grown under normal condition which watered once a day, while another three plants was subjected to soil drought stress by the withdrawal of irrigation, and samples were harvested at 0, 3, 6 and 9 days, respectively. Leaf samples with two ploidy materials were collected for both the ninth day of control and drought stressed samples at four time points; and all materials were performed three replicates, a total of 30 samples, which immediately frozen in liquid nitrogen and stored at − 80 °C for subsequent transcriptome and microRNA sequencing.

#### MicroRNA sequencing

After switchgrass was subjected to drought treatment, a total of 18 samples (three biological replicates) collected at 0, 3, and 6 days of drought stress were used for subsequent miRNA sequencing. At each time point, the leaves were collected from same part of different tillers from same plants.

##### MicroRNA library construction and sequencing

The samples were sent to Tianjin Novogene Co., Ltd. for sequencing. After quality of the RNA samples was determined, the library was constructed using the NEBNext^®^ Multiplex Small RNA Sample Prep Set for Illumina^®^ (NEB, USA.). First, the adapter was directly added to both ends of small RNA, and then the cDNA was synthesized through reverse transcription. PCR amplification were processed using LongAmp Taq 2X Master Mix, SR Primer for illumina and index (X) primer. After PCR amplification, 8% PAGE gel electrophoresis (100 V, 80 min) was used to separate the target DNA fragments, and the cDNA library was obtained after gel cutting and recovery.

The obtained cDNA was diluted to 1 ng/μL and measured by Qubit 2.0. Next, the cDNA library was detected using Agilent 2100 and then accurately quantified by Q-PCR method (the effective concentration of the library is > 2 nM) to ensure the quality of the library. After the library was qualified, the different libraries were pooled according to the effective concentration and the target data volume, and HiSeq/MiSeq sequencing was performed.

##### Identification of conserved and novel miRNAs

Cutadapt-1.2.1 tool and mass fraction pretreatment method were used to preprocess the raw reads of the sequencing, including removing adapters and the low-quality sequence, and then the length distribution diagram of the processed clean reads is made.

The length-screened sRNA (18–30nt) were mapped to the reference sequence by Bowtie, and the reads mapped to the reference sequence are compared with the specified range sequence in the miRBase (Release 20, http://mirbase.org/) to obtain the readcount of the sRNA matched on each sample. Novel miRNAs were predicted using miREvo and mirdeep2 ([90], [91]).

##### MiRNA differential expression analysis

DESeq 2 [92] based on negative binomial distribution was used for miRNA differential expression analysis, and it’s input data is the readcount (the count of reads that mapped on the miRNAs, which are annotated in miRBase database) data of the miRNA. Cluster analysis of differential miRNA is used to judge the clustering pattern of differential miRNA expression under different experimental conditions. The expression of readcount were normalized to transcripts per million (TPM) reads [93]. Formula: normalized expression = (readcount × 1,000,000)/libsize (libsize: sum of samples miRNA readcount). The online analysis platform OmicShare (http://www.omicshare.com/tools) was used for cluster mapping. Comparison of differential expression between groups miRNA Venn diagrams using the online tool VENNY2.1: http://bioinfogp.cnb.csic.es/tools/venny/index.html.

#### RNA-seq

A total of 30 samples collected on 0, 3, 6 and 9 days after drought treatment of two ploidy types as described above were used for transcriptome sequencing.

##### Transcriptome library construction and sequencing

Total RNA was extracted from leaves using TRIzol reagent (Invitrogen, Carlsbad, CA, USA) according to the manufacturer’s procedure. The RNA samples were detected in 4 steps: (1)1% agarose gel electrophoresis is used to analyze the degradation degree of RNA and whether there is pollution; (2) NanoPhotometer spectrophotometer (IMPLEN, CA, USA) to detect RNA purity (OD260/280 ratio); (3) Accurate quantification of RNA concentration using the Qubit kit (Life Technologies, CA, USA); 4) Accurate detection of RNA integrity by Agilent 2100 (Agilent Technologies, CA, USA). Finally, samples with OD260/280 ≥ 1.8, 28S/18S ≥ 1.0, RIN value ≥ 6.3, and RNA concentration ≥ 50 ng/μL were selected as samples for database construction.

After the samples were tested, 3 μg RNA was extracted from each sample as a sequencing input material. The mRNA was enriched by the NEBNext^®^ Poly(A) mRNA Magnetic Isolation Module, and the general transcriptome library was constructed using the recommended program NEBNext^®^ mRNA Library Prep Master Mix Set for Illumina HiSeqTM 4000 with an insert size of approximately 250 bp.

The specific flow is as follows: firstly, magnetic beads with Oligo(dT) are used to enrich and purify mRNA. Then, a fragmentation buffer is added to make mRNA into a short segment, and use the fragment mRNA as a template to synthesize a strand cDNA with random hexamers and RNase H-, then add buffer, dNTPs and DNA polymerase I synthesizes the double-stranded cDNA, and the remaining overhangs are end-repaired by exonuclease/polymerase. The double-stranded cDNA was purified by AMPure XP beads (Beckman Coulter, Beverly, USA), and cDNA fragments of 250–200 bp in length were selected. Finally, PCR amplification was carried out, and the PCR products were purified with AMPure XP beads to obtain the final library. Then the insert size of the library was detected by Agilent 2100, the effective concentration of the library was accurately quantified by Q-PCR method (the effective concentration of the library > 2 nM) to ensure the quality of the library. According to the instructions, the index-coded sample library is clustered on the cBot clustering generation system. Subsequently, different libraries were pooled according to the effective concentration and the target data volume, and sequenced on Illumina Hiseq platform to obtain 125 bp/150 bp double-ended sequences [94, 95].

##### Transcriptome sequencing data analysis

The original sequence was processed by removing reads containing adapters, reads containing poly (N) sequences, and low-quality reads to obtain clean reads. The subsequent analysis is based on high-quality clean reads.

Download the latest version of the switchgrass genomic information (version 4.1) from the JGI data site for reference (https://phytozome.jgi.doe.gov/pz/portal.html#!info?alias=Org_Pvirgatum_er). The reference genome index will be constructed by Bowtie v2.2.3 [96] and then the double-ended clean reads will be aligned to the reference genome using TopHat v2.0.12.

TopHat was chosen as the mapping tool because it can generate a splice point database based on the gene model annotation file, which results in better mapping results than other non-splicing mapping tools([97], [98]).The number of reads aligned to each gene was calculated using HTSeq v0.6.1 [99], and then the FPKM of each gene was calculated according to the length of the gene and the number of reads to the gene. The genomic localization analysis of the filtered sequence was performed using the HISAT software with default parameters.

DESeq 2 [92] in R package was used to analyze differentially expressed genes between different samples, and the parameters were set to adjust *P* value < 0.05, log2FoldChange > 1.

The R software was selected to perform weighted gene co-expression network analysis (WGCNA) on differentially expressed transcripts. WGCNA algorithm is based on the assumed gene network without scale distribution, then constructs gene co-expression correlation matrix and gene network, and defines them as adjacency functions, then analyzes and calculates the dissimilarity coefficients of different nodes to construct hierarchical clustering tree [100]. After obtaining modules with different expression trends, gene annotations were performed on the modules of interest to explore specific molecular functions. Transcripts with a maximum of FPKM less than five in ten sample classifications were selected for filtering [57].

#### Combination analysis of microRNA and transcriptome

PsRNATarget [101] was used to predict the target genes of the screened differentially expressed miRNA. Subsequently, GO and KEGG enrichment analysis was performed on the predicted target genes to explore their functions. Then, the expression level of the corresponding target gene was found by comparing with the transcriptome data. GO and KEGG enrichment analysis and expression mapping were performed using online analysis platform (http://www.omicshare.com/tools). Also by this tool, top enrichment bubble graph was used to show the top enriched terms or pathways for all groups of DET, and the rich factor was calculated using the input gene number against background gene number (all DETs) for every term. The calculated p-value was gone through FDR Correction, taking FDR ≤ 0.05 as a threshold. GO terms meeting this condition were defined as significantly enriched GO terms in DEGs. This analysis was able to recognize the main biological functions that DEGs exercise.

#### Drought-related pathway analysis

PowerPoint 2016 and online platform OmicShare (http://www.omicshare.com/tools) were used to draw the pathway and gene expression, to find gene expression changes in drought-related pathway. Z-score normalization was used here.

## Supplementary information

**Additional file 1: Table S1.** Summary of samples transcriptome sequencing results.

**Additional file 2: Table S2.** Summary of differently expressed transcripts among seven comparisons.

**Additional file 3: Figure S1.** Top 10 enriched GO terms and Top 5 enriched pathways of ploidy-related DETs.

**Additional file 4: Figure S2.** Top 20 enriched GO terms of ploidy- and drought-related DETs in 4X Alamo and 8X Alamo.

**Additional file 5: Table S3.** Summary of samples miRNA sequencing results.

**Additional file 6: Table S4.** Annotation of DEmiR targets.

**Additional file 7: Figure S3.** WGD-related miRNAs’ targets expression.

**Additional file 8.** Protocols of the physiological measurement.

## Data Availability

The raw data of mRNA, microRNA will upload to NCBI.

## Abbreviations

WGD: Whole genome duplication
miRNA: microRNA
DETs: Differently expressed transcripts
WGCNA: Weighted gene co-expression network analysis
GO: Gene ontology
KEGG: Kyoto Encyclopedia of Genes and Genomes
DEmiRs: Differentially expressed microRNAs
SnRK: Sucrose non-fermenting related kinase
SLAC: Slowly activating anion channel
POD: Peroxidase
RWC: Relatively water content
Pro: Free proline
Relative electrical conductivity: REC
Malondialdehyde: MDA
Superoxidase: SOD
Control check: CK
Drought stress: DS
FPKM: Fragments per kilobase of transcript per million fragments mapped
BP: Biological process
MF: Molecular function
cc: Cellular component
TCA: Tricarboxylic acid cycle
UBC: Ubiquitin-conjugating
GDH: Glutamate dehydrogenase
LAC: Laccase
UBQ: Ubiquitin
GLR: Glutamate Receptor
CSD: Cu.Zn—SOD
LEA: Late Embryogenesis Abundant Proteins
ATPD: ATP synthase delta subunit gene
CYP: Cytochrome
CCS: Copper chaperone protein
PP2C: Type 2C protein phosphatase
DREB: Dehydration responsive element binding protein
