# Supplementary material for: Comparative transcriptome study of switchgrass (Panicum virgatum L.) homologous autopolyploid and its parental amphidiploid responding to consistent drought stress
Source: Biotechnol Biofuels. 2020 Oct 15;13:170. doi: 10.1186/s13068-020-01810-z (PMC7559793; doi:10.1186/s13068-020-01810-z)
Supplement: Supplementary file 1 — Additional file 1: Table S1. Summary of samples transcriptome sequencing results. [file 13068_2020_1810_MOESM1_ESM.docx]

**Additional file 1**

**Table S1 Summary of samples transcriptome sequencing results**

| Sample name | Raw reads | Clean reads | clean bases | Error rate  (%) | Q20  (%) | Q30  (%) | GC content  (%) |
| --- | --- | --- | --- | --- | --- | --- | --- |
| CK4_1 | 54654366 | 47429910 | 7.11G | 0.02 | 96.95 | 92.14 | 58.28 |
| CK4_2 | 43857314 | 40702728 | 6.11G | 0.02 | 96.22 | 90.95 | 58.87 |
| CK4_3 | 44583956 | 41463186 | 6.22G | 0.02 | 96.33 | 91.18 | 58.71 |
| CK8_1 | 46138050 | 43120318 | 6.47G | 0.02 | 96.36 | 91.22 | 59.54 |
| CK8_2 | 44231510 | 41644716 | 6.25G | 0.02 | 96.38 | 91.27 | 57.62 |
| CK8_3 | 50015620 | 46255052 | 6.94G | 0.02 | 96.22 | 90.95 | 60.32 |
| DS4_3_1 | 50409410 | 47218998 | 7.08G | 0.02 | 96.39 | 91.30 | 59.32 |
| DS4_3_2 | 52261598 | 49163580 | 7.37G | 0.02 | 96.39 | 91.54 | 58.95 |
| DS4_3_3 | 60636020 | 56536610 | 8.48G | 0.02 | 96.61 | 91.99 | 55.19 |
| DS8_3_1 | 53552730 | 50285226 | 7.54G | 0.02 | 96.30 | 91.41 | 57.57 |
| DS8_3_2 | 68628860 | 64461544 | 9.67G | 0.02 | 96.21 | 91.25 | 58.92 |
| DS8_3_3 | 58379808 | 54399672 | 8.16G | 0.02 | 96.51 | 91.81 | 59.38 |
| DS4_6_1 | 64047798 | 60398128 | 9.06G | 0.02 | 96.39 | 91.57 | 58.98 |
| DS4_6_2 | 50999210 | 46972432 | 7.05G | 0.02 | 96.36 | 91.54 | 58.48 |
| DS4_6_3 | 62566654 | 59063792 | 8.86G | 0.01 | 97.30 | 93.21 | 56.75 |
| DS8_6_1 | 59189872 | 54983140 | 8.25G | 0.02 | 96.60 | 91.90 | 58.49 |
| DS8_6_2 | 55667328 | 51995548 | 7.8G | 0.02 | 96.50 | 91.70 | 59.20 |
| DS8_6_3 | 58781960 | 54886084 | 8.23G | 0.02 | 96.60 | 91.89 | 58.03 |
| DS4_9_1 | 60602314 | 56578470 | 8.49G | 0.02 | 96.39 | 91.52 | 57.99 |
| DS4_9_2 | 59899718 | 56046932 | 8.41G | 0.02 | 96.48 | 91.69 | 57.80 |
| DS8_9_1 | 54021556 | 50155334 | 7.52G | 0.02 | 96.52 | 91.76 | 60.16 |
| DS8_9_2 | 45858108 | 42764936 | 6.41G | 0.02 | 96.23 | 91.00 | 58.62 |
| DS8_9_3 | 67590950 | 62049922 | 9.31G | 0.02 | 96.68 | 91.66 | 58.83 |
| CK4_9_1 | 58294014 | 54541988 | 8.18G | 0.02 | 96.52 | 91.73 | 58.25 |
| CK4_9_2 | 50480198 | 47677950 | 7.15G | 0.02 | 97.05 | 92.66 | 61.67 |
| CK4_9_3 | 57318066 | 52388058 | 7.86G | 0.01 | 97.35 | 93.34 | 59.64 |
| CK8_9_1 | 61473000 | 57246460 | 8.59G | 0.01 | 97.32 | 93.24 | 59.29 |
| CK8_9_2 | 63817258 | 58886192 | 8.83G | 0.01 | 97.33 | 93.26 | 58.16 |
| CK8_9_3 | 69476368 | 65443934 | 9.82G | 0.02 | 97.10 | 92.83 | 59.50 |
