# Supplementary material for: Comparative transcriptome study of switchgrass (Panicum virgatum L.) homologous autopolyploid and its parental amphidiploid responding to consistent drought stress
Source: Biotechnol Biofuels. 2020 Oct 15;13:170. doi: 10.1186/s13068-020-01810-z (PMC7559793; doi:10.1186/s13068-020-01810-z)
Supplement: Supplementary file 2 — Additional file 2: Table S2. Summary of differently expressed transcripts among seven comparisons. [file 13068_2020_1810_MOESM2_ESM.docx]

Additional file 2:

**Table S2 Summary of differently expressed transcripts among seven comparisons**

| comparisons | | Number of up-regulated transcripts | | Number of down-regulated transcripts | | Number of differently expressed transcripts | |
| --- | --- | --- | --- | --- | --- | --- | --- |
| DS4_9vsCK4_9 | | 7921 | | 8736 | | 16657 | |
| DS8_9vsCK8_9 | | 10522 | | 10384 | | 20906 | |
| CK8vsCK4 | | 6375 | | 6253 | | 12628 | |
| CK8_9vsCK4_9 | | 6163 | | 6101 | | 12264 | |
| DS8_3vsDS4_3 | | 2421 | | 2000 | | 4421 | |
| DS8_6vsDS4_6 | | 4693 | | 4661 | | 9354 | |
| DS8_9vsDS4_9 | | 4625 | | 3010 | | 7635 | |
