# Supplementary figures and images for: Comparative transcriptome study of switchgrass (Panicum virgatum L.) homologous autopolyploid and its parental amphidiploid responding to consistent drought stress
Source: Biotechnol Biofuels. 2020 Oct 15;13:170. doi: 10.1186/s13068-020-01810-z (PMC7559793; doi:10.1186/s13068-020-01810-z)

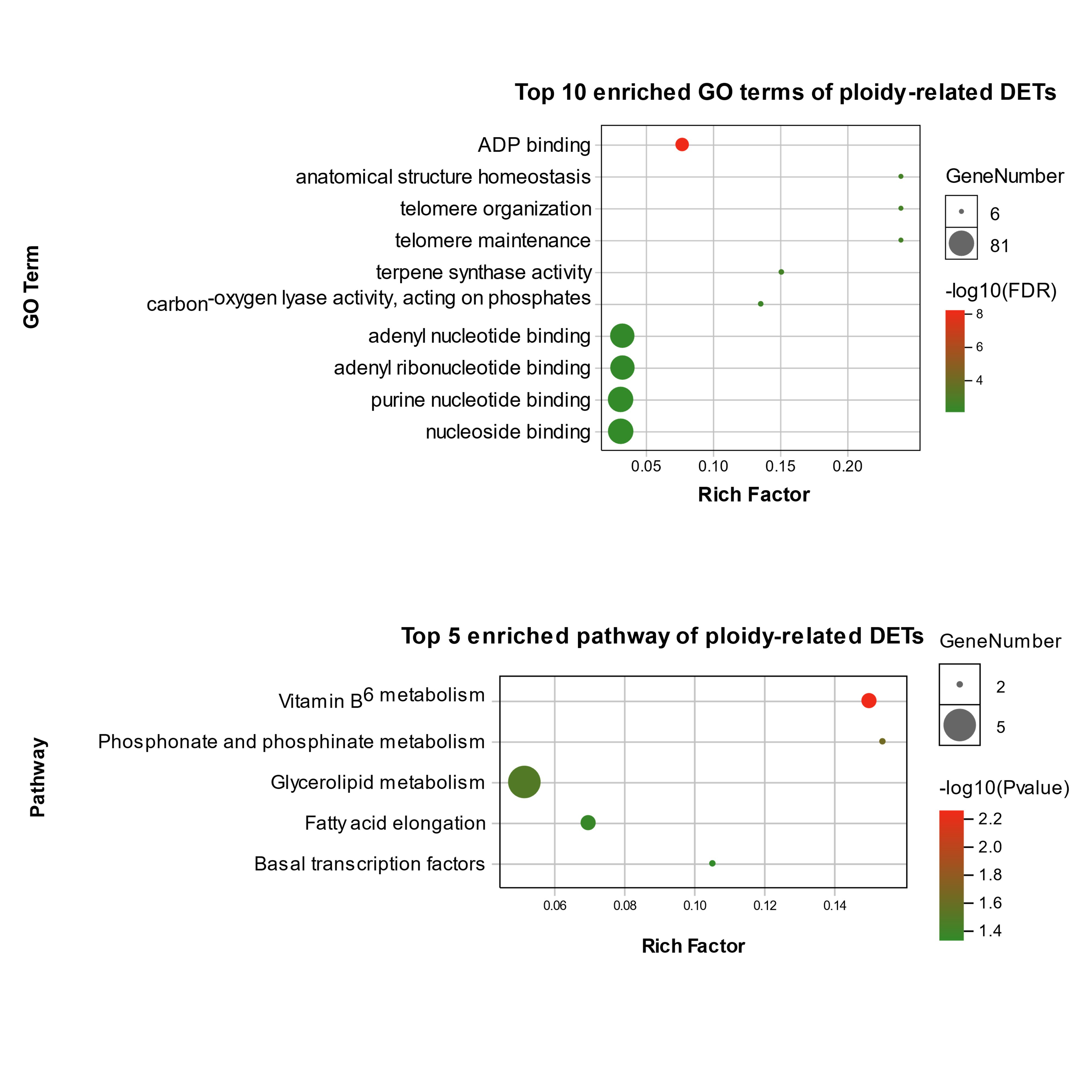

Supplement: Supplementary file 3 — Additional file 3: Figure S1. Top 10 enriched GO terms and Top 5 enriched pathways of ploidy-related DETs. [file 13068_2020_1810_MOESM3_ESM.tif]

**sbi-miR397-3p**

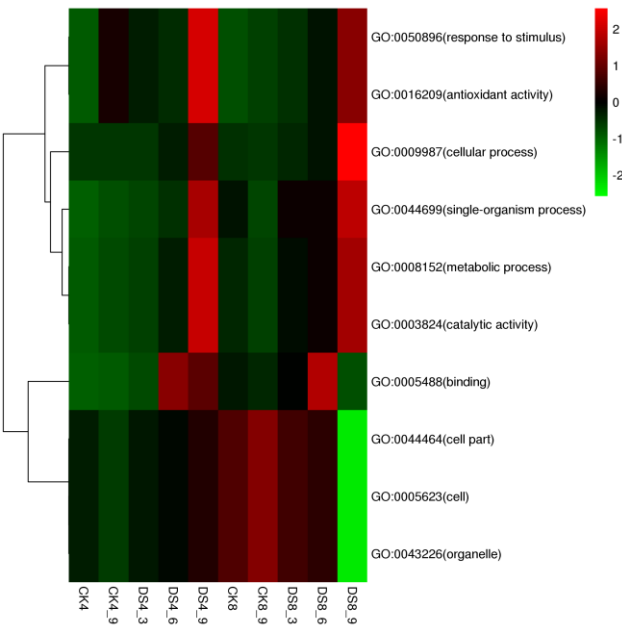

**sbi-miR399d**

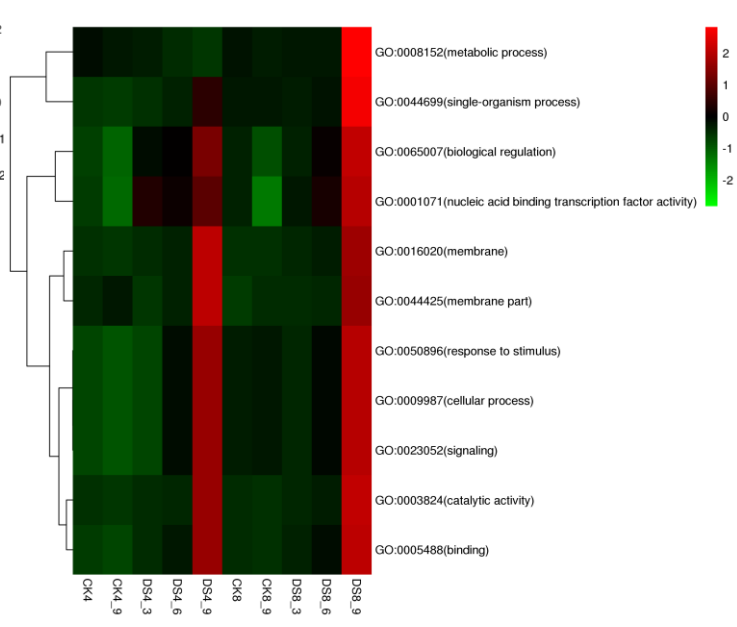

**sbi-miR397-5p**

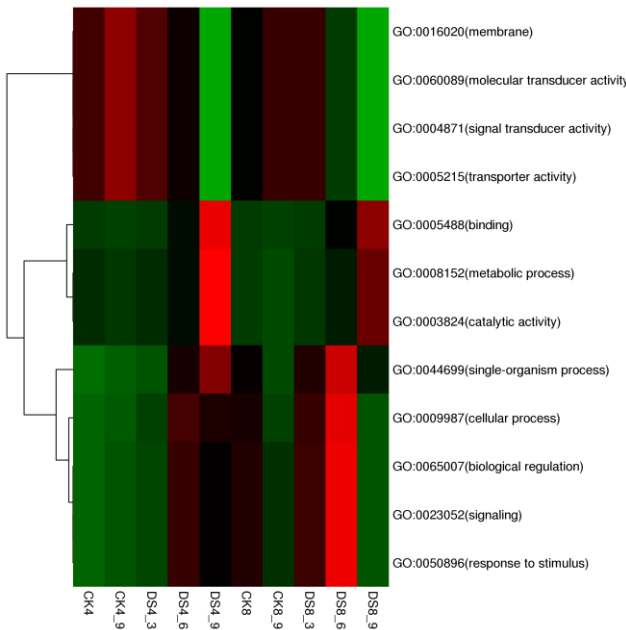

**sbi-miR528**

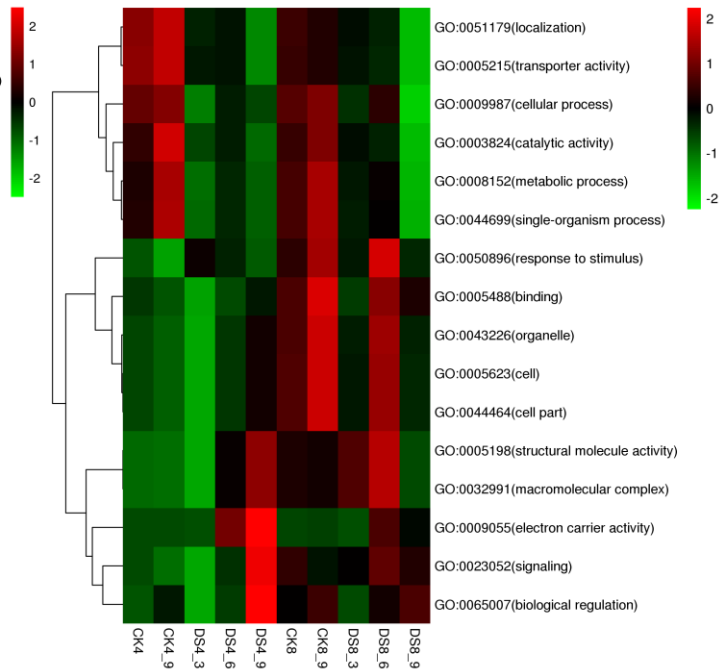

Supplement: Supplementary file 7 — Additional file 7: Figure S3. WGD-related miRNAs’ targets expression. [file 13068_2020_1810_MOESM7_ESM.pdf]
