# Supplementary material for: Comparative transcriptome study of switchgrass (Panicum virgatum L.) homologous autopolyploid and its parental amphidiploid responding to consistent drought stress
Source: Biotechnol Biofuels. 2020 Oct 15;13:170. doi: 10.1186/s13068-020-01810-z (PMC7559793; doi:10.1186/s13068-020-01810-z)
Supplement: Supplementary file 4 — Additional file 4: Figure S2. Top 20 enriched GO terms of ploidy- and drought-related DETs in 4X Alamo and 8X Alamo. [file 13068_2020_1810_MOESM4_ESM.pdf]

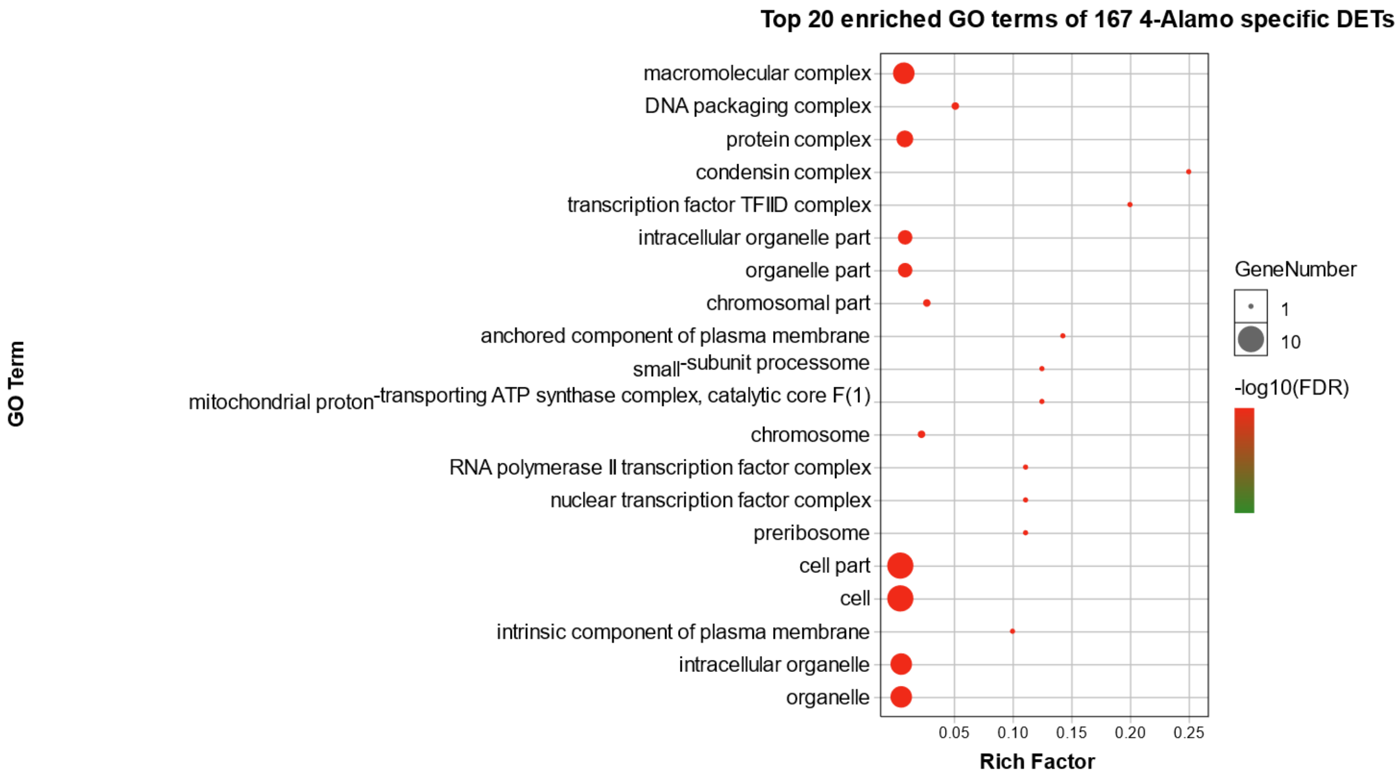
Top 20 enriched GO terms of 167 4-Alamo specific DETs
macromolecular complex
DNA packaging complex
protein complex
condensin complex
transcription factor TFIID complex
intracellular organelle part
organelle part
chromosomal part
anchored component of plasma membrane
small-subunit processome
mitochondrial proton-transporting ATP synthase complex, catalytic core F(1)
chromosome
RNA polymerase II transcription factor complex
nuclear transcription factor complex
preribosome
cell part
cell
intrinsic component of plasma membrane
intracellular organelle
organelle
GO Term
0.05
0.10
0.15
0.20
0.25
Rich Factor
GeneNumber
1
10
-log10(FDR)

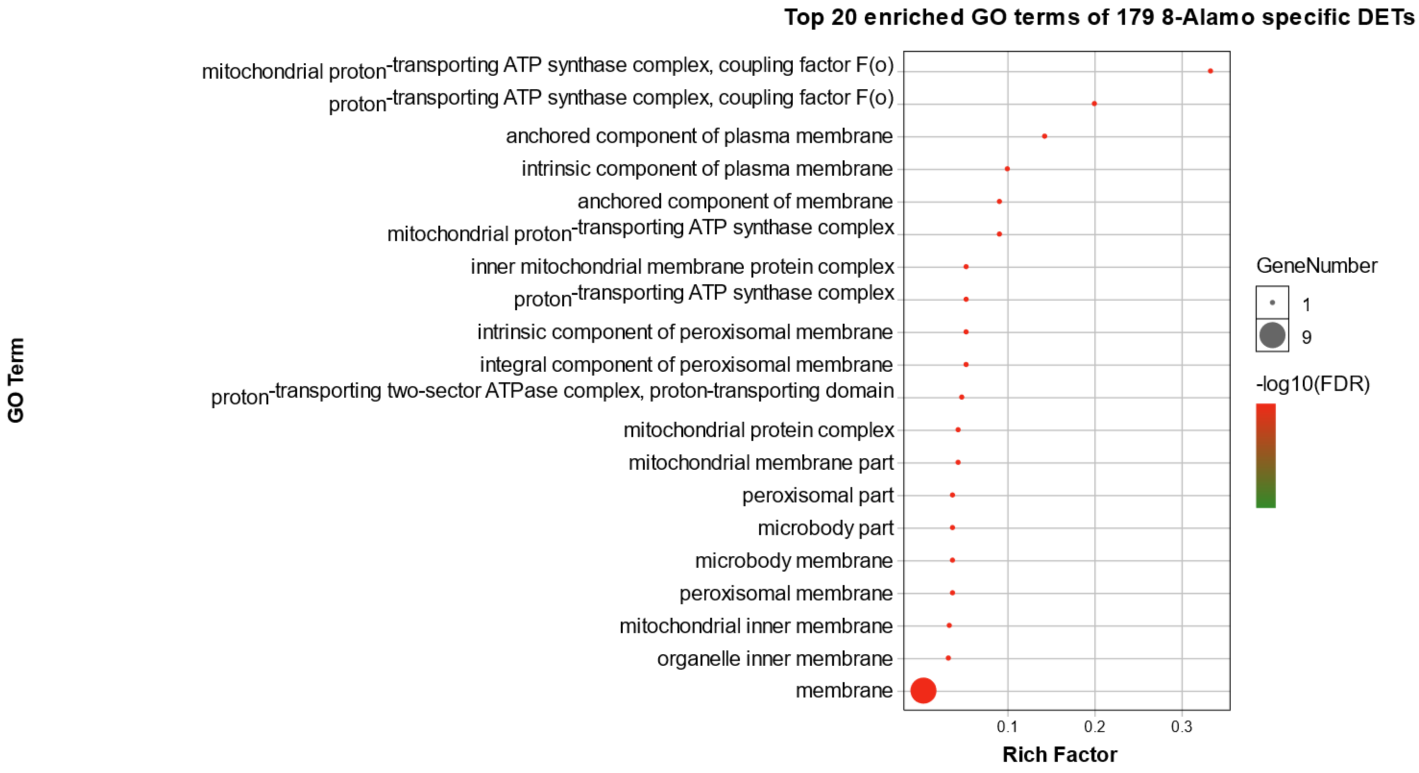
Top 20 enriched GO terms of 179 8-Alamo specific DETs
mitochondrial proton-transporting ATP synthase complex, coupling factor F(o)
proton-transporting ATP synthase complex, coupling factor F(o)
anchored component of plasma membrane
intrinsic component of plasma membrane
anchored component of membrane
mitochondrial proton-transporting ATP synthase complex
inner mitochondrial membrane protein complex
proton-transporting ATP synthase complex
intrinsic component of peroxisomal membrane
integral component of peroxisomal membrane
proton-transporting two-sector ATPase complex, proton-transporting domain
mitochondrial protein complex
mitochondrial membrane part
peroxisomal part
microbody part
microbody membrane
peroxisomal membrane
mitochondrial inner membrane
organelle inner membrane
membrane
GO Term
0.1
0.2
0.3
Rich Factor
GeneNumber
1
9
-log10(FDR)
