# Supplementary material for: Comparative transcriptome study of switchgrass (Panicum virgatum L.) homologous autopolyploid and its parental amphidiploid responding to consistent drought stress
Source: Biotechnol Biofuels. 2020 Oct 15;13:170. doi: 10.1186/s13068-020-01810-z (PMC7559793; doi:10.1186/s13068-020-01810-z)
Supplement: Supplementary file 5 — Additional file 5: Table S3. Summary of samples miRNA sequencing results. [file 13068_2020_1810_MOESM5_ESM.docx]

Additional file 5:

**Table S3 Summary of samples miRNA sequencing results**

| Sample | Reads | Bases | Error rate | Q20 | Q30 | GC content |
| --- | --- | --- | --- | --- | --- | --- |
| CK4_1 | 13143251 | 0.657G | 0.01% | 97.16% | 93.76% | 50.17% |
| CK4_2 | 12815820 | 0.641G | 0.01% | 96.99% | 93.38% | 50.60% |
| CK4_3 | 12721487 | 0.636G | 0.01% | 96.72% | 92.78% | 49.92% |
| CK8_1 | 12401547 | 0.620G | 0.01% | 97.10% | 93.59% | 50.15% |
| CK8_2 | 12460931 | 0.623G | 0.01% | 97.15% | 93.69% | 50.11% |
| CK8_3 | 13432903 | 0.672G | 0.01% | 97.06% | 93.54% | 49.91% |
| DS4_3_1 | 13117697 | 0.656G | 0.01% | 97.29% | 94.02% | 50.54% |
| DS4_3_2 | 13381140 | 0.669G | 0.01% | 97.27% | 94.00% | 49.84% |
| DS4_3_3 | 13539858 | 0.677G | 0.01% | 97.11% | 93.77% | 50.06% |
| DS8_3_1 | 13753506 | 0.688G | 0.01% | 97.08% | 93.57% | 49.81% |
| DS8_3_2 | 14222797 | 0.711G | 0.01% | 96.94% | 93.32% | 50.36% |
| DS8_3_3 | 12247569 | 0.612G | 0.01% | 96.89% | 93.38% | 49.86% |
| DS4_6_1 | 14947875 | 0.747G | 0.01% | 97.03% | 93.51% | 50.44% |
| DS4_6_2 | 13723443 | 0.686G | 0.01% | 96.99% | 93.40% | 50.57% |
| DS4_6_3 | 11239826 | 0.562G | 0.01% | 97.27% | 94.03% | 49.85% |
| DS8_6_1 | 14400900 | 0.720G | 0.01% | 96.94% | 93.34% | 50.33% |
| DS8_6_2 | 13376506 | 0.669G | 0.01% | 97.02% | 93.50% | 50.14% |
| DS8_6_3 | 13488268 | 0.674G | 0.01% | 97.37% | 94.18% | 50.41% |
