# Supplementary material for: Comparative transcriptome study of switchgrass (Panicum virgatum L.) homologous autopolyploid and its parental amphidiploid responding to consistent drought stress
Source: Biotechnol Biofuels. 2020 Oct 15;13:170. doi: 10.1186/s13068-020-01810-z (PMC7559793; doi:10.1186/s13068-020-01810-z)
Supplement: Supplementary file 8 — Additional file 8. Protocols of the physiological measurement. [file 13068_2020_1810_MOESM8_ESM.docx]

**Additional file 8: Protocols of the physiological measurement**

Random leaves in the middle part of each plants were collected to process following measurement.

**Chlorophyll content:**

Leaves were ground in a solution of 1 part 0.1 Normal (N) ammonium hydroxide solution to 9 parts 80% acetone [volume to volume (v:v)]. The mixture is centrifuged, and then the supernatant is diluted to a concentration that absorbance reading is between 0.2 and 0.8 at wavelengths of 663 nanometers (nm) and 645 nm. The absorbance of each solution is recorded and the concentrations of chlorophyll a and b are calculated according to following formula:

Chlorophyll a [milligrams/milliliter (mg/mL)] = 12.7 A_663_ - 2.69 A_645_

Chlorophyll b (mg/mL) = 22.9 A_645_ - 4.68 A_663_

Total Chlorophyll (mg/mL) = Chlorophyll a + Chlorophyll b.

**Relative water content (RWC):**

To determine RWC the second fully opened leaves were sampled. The leaves were collected in falcon tubes and transported to the laboratory as quickly as possible to minimize water losses. Next, the samples were weighed immediately as fresh weight (FW), then floated on distilled water for 4 h at 4 ^o^C. The leaves were then rapidly blotted to remove surface water and weighed to obtain turgid weight (TW). Lastly, the samples were dried in the oven at 60 ^o^C for 24 h and dry weight (DW) were obtained.

The RWC was calculated by the following formula: RWC (%) = [FW-DW)/(TW-DW] * 100

**REC:**

The electrolyte leakage test was performed as reported by Yu et al.

(2006). Briefly, washed leaves were cut into 1 cm slices and put

into a test tube containing 5 ml of deionized water. The leaf

samples were immersed and vibrated occasionally at 258C for

2 h, and then the electrical conductivity of the solution (C1)

was measured. After boiling the samples for 10 min, the

conductivity (C2) was measured again after the solution was

cooled to room temperature. The REC was calculated as follows:

REC (%) ¼C1/C2 100

The electrolyte leakage test was performed as reported by Yu et al.

(2006). Briefly, washed leaves were cut into 1 cm slices and put

into a test tube containing 5 ml of deionized water. The leaf

samples were immersed and vibrated occasionally at 258C for

2 h, and then the electrical conductivity of the solution (C1)

was measured. After boiling the samples for 10 min, the

conductivity (C2) was measured again after the solution was

cooled to room temperature. The REC was calculated as follows:

REC (%) ¼C1/C2 100

The electrolyte leakage test was performed as reported by Yu et al.

(2006). Briefly, washed leaves were cut into 1 cm slices and put

into a test tube containing 5 ml of deionized water. The leaf

samples were immersed and vibrated occasionally at 258C for

2 h, and then the electrical conductivity of the solution (C1)

was measured. After boiling the samples for 10 min, the

conductivity (C2) was measured again after the solution was

cooled to room temperature. The REC was calculated as follows:

REC (%) ¼C1/C2 100

The Relative electrical conductivity was measured as reported by Yu et al.(2006). Washed leaves were cut into 1 cm slices and put into a falcon tube containing 5 ml of deionized water. The samples were immersed and vibrated at the same time for 2 h at 25 ^o^C, and then the electrical conductivity of the solution (C1) was measured by a conductivity meter. After boiling the samples for 10 min and cooled down to room temperature, the conductivity (C2) was measured again.

The REC was calculated as: REC (%) = C1/C2 *100

**Malondialdehyde (MDA) content**:

1. Extraction of MDA:
2. Weigh 0.2g of leaves, shred it and put it in a mortar.
3. Add 1ml of trichloroacetic acid (TCA) and quartz sand, grind it into a homogenate and transfer it to a 10ml centrifuge tube, and wash the mortar with TCA, the washing liquid is also transferred into the centrifuge tube. The total amount of TCA should be 7 ml.
4. Centrifuge the homogenate at 4500 rpm for 20 min, and transfer the supernatant to a 10 ml clean centrifuge tube adding distilled water to 10 ml to get the extract.
5. Color reaction:
6. Add 3ml of extract (add 3ml of distilled water as blank) to a 10ml calibrated test tube.
7. Add 0.5% thiobarbituric acid (TBA) 3ml to each tube, shake well
8. Water bath in boiling water for 15min (start counting when small bubbles appear in the solution), then immediately put the tubes in cold water.

3. Colorimetric: With blank as reference, the extinction values of the sample solutions are measured at 450nm, 532nm and 600nm with a spectrophotometer.

Calculation:

C: MDA concentration in the extract (μmol·L^-1^)

V: total volume of extract (ml)

W: fresh weight of sample (g)

C (μmol·L^-1^) = 6.45 (OD_532_-OD_600_) -0.56 OD_450_

MDA content (μmol·g^-1^) = (C×V×10^-3^)/W

**Peroxidase (POD) activity**:

The activity of POD was measured using guaiacol oxidation method (Chance and Maehly, 1955).

1. The reaction solution (3 mL) contained:

2.7 ml of 50 mM phosphate buffer (pH 7.0)

0.1 ml of 1.5% guaiacol

0.1 ml of 300 mM H2O2

0.1 ml of enzyme extract

A solution containing distilled water in place of enzyme was used as the control.

1. Changes in absorbance of the reaction solution, due to guaicol oxidation, was measured at 470 nm by a spectrophotometer over one-minute time scan.
2. The activity of POD was expressed as U mg^-1^ protein per minute where U represents the extension of mmoles units (U) of guaiacol.

**Superoxide Dismutases (SOD) activity**:

SOD activity was determined by the method of Giannopolitis and Ries.

1. The final volume (1 ml) of reaction mixture in test tubes contained:

50 mM phosphate buffer (pH 7.8)

0.1 mM EDTA

13 mM methionine

65 μM NBT

1.3 μM riboflavin

30 μL SOD extract.

A test tube containing all reacting solutions and distilled water in place of enzyme extract served as blank which was used as reference

1. Irradiate test tubes under fluorescent lights 60 μmol·m^-2^·s^-1^ for 10 min at room temperature (25 ^o^C). The absorbances of the sample solutions are measured at 560 nm by a spectrophotometer.
2. Enzyme activity unit (U): the amount of enzyme when inhibits 50% of NBT photoreduction.
